# Bidirectional promoters are the major source of gene activation-associated non-coding RNAs in mammals

**DOI:** 10.1186/1471-2164-15-35

**Published:** 2014-01-17

**Authors:** Masahiro Uesaka, Osamu Nishimura, Yasuhiro Go, Kinichi Nakashima, Kiyokazu Agata, Takuya Imamura

**Affiliations:** 1 Department of Biophysics and Global COE Program, Graduate School of Science, Kyoto University, Kitashirakawa-Oiwake, Sakyo-ku, Kyoto 606-8502, Japan; 2 Department of Stem Cell Biology and Medicine, Graduate School of Medical Sciences, Kyushu University, 3-1-1 Maidashi, Higashi-ku, Fukuoka 812-8581, Japan; 3 Genome Resource and Analysis Unit, RIKEN Center for Developmental Biology, 2-2-3 Minatojima-Minamimachi, Chuo-ku, Kobe 650-0047, Japan; 4 Department of Cellular and Molecular Biology, Primate Research Institute, Kyoto University, 41-2 Kanrin, Inuyama, Aichi 484-8506, Japan; 5 Present address: Department of Brain Sciences, Center for Novel Science Initiatives, National Institutes of Natural Sciences, 38 Nishigonaka Myodaiji, Okazaki, Aichi 444-8585, Japan

**Keywords:** Bidirectional promoter, Non-coding RNA, CpG island, Directional RNA-Seq, Gene activation

## Abstract

**Background:**

The majority of non-coding RNAs (ncRNAs) involved in mRNA metabolism in mammals have been believed to downregulate the corresponding mRNA expression level in a pre- or post-transcriptional manner by forming short or long ncRNA-mRNA duplex structures. Information on non-duplex-forming long ncRNAs is now also rapidly accumulating. To examine the directional properties of transcription at the whole-genome level, we performed directional RNA-seq analysis of mouse and chimpanzee tissue samples.

**Results:**

We found that there is only about 1% of the genome where both the top and bottom strands are utilized for transcription, suggesting that RNA-RNA duplexes are not abundantly formed. Focusing on transcription start sites (TSSs) of protein-coding genes revealed that a significant fraction of them contain switching-points that separate antisense- and sense-biased transcription, suggesting that head-to-head transcription is more prevalent than previously thought. More than 90% of head-to-head type promoters contain CpG islands. Moreover, CCG and CGG repeats are significantly enriched in the upstream regions and downstream regions, respectively, of TSSs located in head-to-head type promoters. Genes with tissue-specific promoter-associated ncRNAs (pancRNAs) show a positive correlation between the expression of their pancRNA and mRNA, which is in accord with the proposed role of pancRNA in facultative gene activation, whereas genes with constitutive expression generally lack pancRNAs.

**Conclusions:**

We propose that single-stranded ncRNA resulting from head-to-head transcription at GC-rich sequences regulates tissue-specific gene expression.

## Background

Protein-coding regions account for only about 1.5% of the human genome [1], but the FANTOM Consortium and the ENCODE Project Consortium revealed that more than 62% of the genomic DNA acts as a template for transcription [2,3], indicating that there are a large number of non-coding RNAs (ncRNAs) in living cells. Recently, many functional ncRNAs have been identified. It is well known that small RNAs, such as miRNAs and piRNAs, act in post-transcriptional regulation by forming RNA-RNA duplexes [4,5]. In addition to these RNAs, many kinds of long ncRNAs have been shown to function in post-transcriptional regulation, such as RNA editing, splicing and translation, by forming RNA-RNA duplexes [6-13]. Indeed, 4,520 sense-antisense transcript (SAT) pairs in mice have the potential to form RNA-RNA duplexes [14]. RNA-RNA duplexes also play a role in transcriptional gene silencing through DNA methylation and histone modifications [15-18]. Thus, it is clear that the formation of RNA-RNA duplexes is important for the mRNA silencing triggered by ncRNA.

However, several studies have reported that some long ncRNAs cause transcriptional activation of genes without forming RNA-RNA duplexes. For example, *HOTTIP*, a long intergenic ncRNA (lncRNA) transcribed from the 5’-end of the *HOXA* locus, binds to an adaptor protein, WD repeat-containing protein 5 (WDR5), which in turn recruits the mixed-lineage leukaemia (MLL) histone methyltransferase complex [19]. With the help of *HOTTIP*-WDR5-MLL1 interaction, several distantly located target genes are brought into close contact through tertiary structure formation, resulting in trimethylation of histone H3K4 and gene activation. Moreover, a recent study showed that *DBE-T*, a chromatin-associated ncRNA, is selectively transcribed from the chromosome 4q35 region in facioscapulohumeral muscular dystrophy patients and coordinates the transcription of 4q35 genes [20]. *DBE-T* recruits the Trithorax group protein ASH1L, a histone-lysine N-methyltransferase, to the DNA template for *DBE-T*, driving histone H3K36 dimethylation and 4q35 gene transcription. Therefore, lncRNAs acting together with chromosomal proteins are thought to regulate gene functions in an RNA-RNA hybridization-independent manner. However, in contrast to small RNAs, there are few reports about the functional properties of single-strand ncRNAs that act without forming RNA-RNA duplexes.

In mammals, CpG islands (CGIs) in promoter regions tend to show bidirectional promoter activity [21,22]. CGIs are utilized for bidirectional transcription in a head-to-head (HtH) manner. Our previous reports have shown that, in contradiction to the prevalent idea that ncRNAs other than classical ncRNAs (tRNA, rRNA, snRNA and snoRNA) downregulate target gene expression, antisense long ncRNAs derived from promoter regions of their respective protein-coding genes activate the expression of those genes via sequence-specific DNA demethylation [23,24]. We termed these antisense long (>200 nt) ncRNAs “promoter-associated ncRNAs” (pancRNAs). At present, little is known about the concerted expression of mRNAs and antisense transcripts produced in their 5’-flanking regions, and comprehensive transcriptome analysis focusing on the bidirectional transcription of mRNA and pancRNA has not been performed. We do not yet know the sequence characteristics of bidirectionally transcribed promoter regions. Here, we examine whether there is a correlation between the expression of sense and antisense transcripts at the genome-wide level using directional RNA-seq. We map the origin of the sense and antisense transcripts found by directional RNA-seq to determine the prevalence of HtH transcript pairs from CGI promoters. We propose that highly expressed antisense transcripts derived from bidirectional transcription start sites (TSSs) show coordinated transcription with the corresponding protein-coding genes.

## Results

### Both top and bottom strands are utilized in a small fraction of the genome

**Figure 1:**
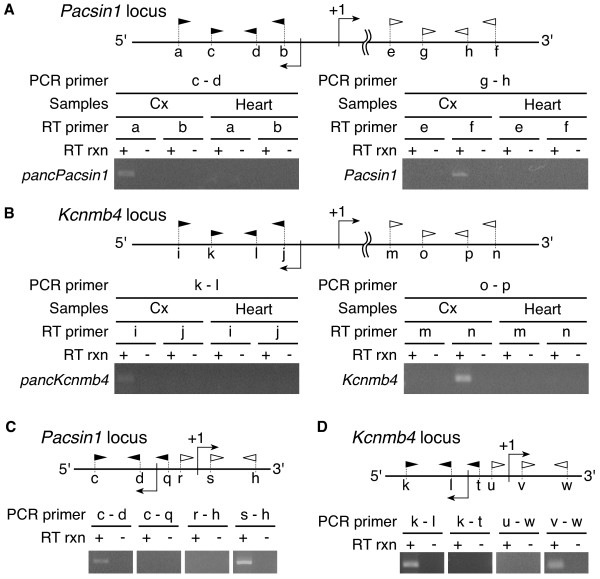
**Determination of the transcriptional direction of pancRNAs and mRNAs.** At the top of each panel, there is a schematic representation of promoter regions of *Pacsin1 ***(A)(C)** and *Kcnmb4 ***(B)(D)**. Filled and open arrowheads represent the primer sets used for the strand-specific RT-PCR analysis for expression of pancRNAs and mRNAs, respectively. Detailed primer information is given in Additional file 1: Table S9. **(A)(B)** Strand-specific RT-PCR analysis for expression of mRNA and pancRNAs in the mouse cerebral cortex (Cx) and heart (Heart). **(C)(D)** RT-PCR analysis for expression of mRNA and pancRNAs in the mouse cerebral cortex. + and - mean the use and lack of reverse transcriptase in the reverse transcription (RT rxn).

We analyzed directional RNA-seq data in order to distinguish sense and antisense transcripts in the mouse cerebral cortex, cerebellum and heart, and in the chimpanzee cerebral cortex and cerebellum [DDBJ:DRA000860]. On average, we obtained 76.3 ± 1.3 million and 228.3 ± 10.7 million reads per sample from the first and second runs of Illumina HiSeq 2000, respectively (Additional file 1: Table S1). The average number of reads passing the read trimming was 72.1 ± 1.2 million and 198.3 ± 9.1 million for the first and second runs, respectively. We mapped the valid reads onto the reference genome sequences using TopHat (see Methods). We used the human instead of the chimpanzee genome as a reference for the chimpanzee reads (see Discussion for the reason). The average percentage of uniquely mapped reads in the valid reads was 86.1% for the mouse cerebral cortex, 85.4% for the mouse cerebellum, 72.6% for the mouse heart, 78.1% for the chimpanzee cerebral cortex and 82.0% for the chimpanzee cerebellum (Additional file 1: Table S1, S2). After we removed duplicate sequences, the average number of uniquely mapped reads in two replicates of each tissue sample was 19.2 million reads for the mouse cerebral cortex, 30.3 million reads for the mouse cerebellum, 18.1 million reads for the mouse heart, 19.0 million reads for the chimpanzee cerebral cortex and 22.5 million reads for the chimpanzee cerebellum. Removal of duplicated reads smoothed the unexpected protruding clusters of reads, possibly derived from PCR bias during library preparation, as shown in Additional file 2: Figure S1. The average ratio of top strand-mapped reads to bottom strand-mapped reads in a sample was 1.0 (Additional file 2: Figure S2 and Additional file 1: Table S1). We confirmed that candidate pancRNAs at *Pacsin1* and *Kcnmb4* (*pancPacsin1* and *pancKcnmb4*) were transcribed from the opposite DNA strand compared to their mRNAs, as expected, using strand-specific RT-PCR (Figure 1A, B). Although we did not confirm the functionality of the candidate pancRNAs, we refer to these transcripts as a fraction of ncRNAs based on their lower coding potential as explained later (Additional file 2: Figure S5A, B). The results of the RT-PCR analysis support the validity of our directional RNA-seq analyses. Then, we calculated the reads per kilobase per million mapped reads (RPKM) of protein-coding genes in the two replicates in order to confirm the reproducibility of our analysis. The Kendall’s tau correlation between the two replicates of each tissue sample was > 0.96 (p < 2.2e^−16^). Therefore, we merged the data from these two replicates for all samples and used them for the following analyses.

In the cerebral cortex, cerebellum and heart, the transcribed regions for polyA+ RNA were found to account for 25.0%, 30.0% and 21.6% of the mouse genome, respectively (Table 1). Next, we examined how many genomic regions were utilized for both sense and antisense transcription. Overlapping transcription for polyA+ RNAs was found in only 0.7%, 1.3% and 0.7% of the mouse genome in the cerebral cortex, cerebellum and heart, respectively (Table 1). A similar transcriptional landscape was found when chimpanzee samples were analyzed (Additional file 1: Table S3).

**Table 1:** The percentage of transcribed regions in the whole genome

	**Transcribed regions**	**Unidirectionally transcribed regions**^**a**^	**Bidirectionally transcribed regions**^**b**^
Cerebral cortex	25.0%	24.3%	0.7%
Cerebellum	30.0%	28.7%	1.3%
Heart	21.6%	20.9%	0.7%

^a^Regions where either sense or antisense transcripts (but not both) originated.

^b^Regions where both sense and antisense transcripts originated.

We calculated the ratio of top strand-mapped reads to bottom strand-mapped reads in the bidirectionally transcribed regions (Additional file 2: Figure S3A, B, C). The results showed that, even if the regions are bidirectionally transcribed, most of the regions show a biased expression pattern in terms of directional transcription. The mapping information in the bidirectionally transcribed regions was subgrouped into top strand, bottom strand and intergenic regions of mouse genes (Additional file 2: Figure S3F, G, H). Significantly large fractions of the top strand- and bottom strand-mapped reads were thereby confirmed to be associated with the top and bottom strands of mouse genes, respectively. In intergenic regions, we also found biased transcription in terms of the directionality. Similarly, biased transcription was also found when chimpanzee samples were analyzed (Additional file 2: Figure S3D, E, I, J).

Taken together, these data showed that strand bias of transcription occurred on a genome-wide level. Either top or bottom strand was preferentially utilized depending on the tissue.

### Genome-wide production of ncRNAs that do not form RNA-RNA duplexes

**Figure 2:**
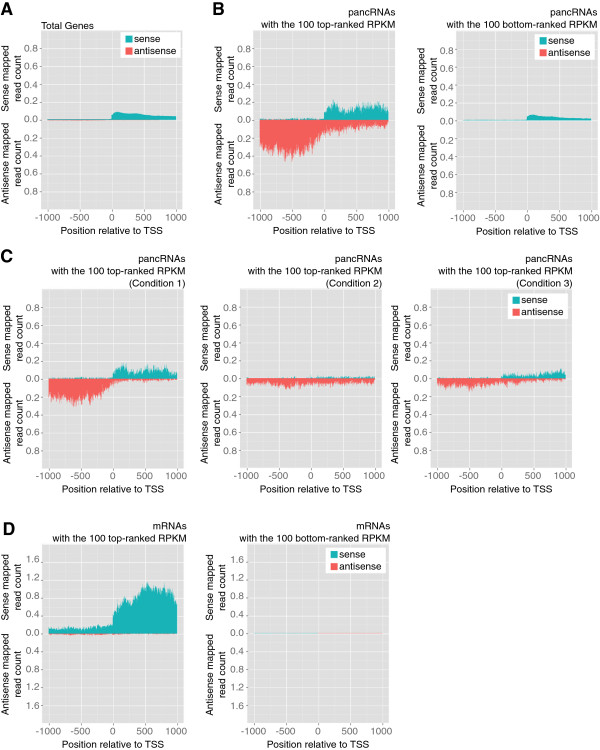
**The expression of pancRNAs showed a positive correlation with that of the corresponding mRNAs.** The distribution of sense and antisense mapped reads around the TSS of each gene fraction in the mouse cerebral cortex. The values in this figure are normalized by the number of genes. **(A)** All reference genes. **(B)** The genes with the 100 most highly expressed pancRNAs (left) and with the 100 most weakly expressed pancRNAs (right), as indicated by RPKM. **(C)** The distribution in Figure 2B was divided into three groups: the genes whose antisense transcript expression level from the upstream region of the TSS was five times higher than that from the downstream region (Condition 1; Left panel). The genes whose antisense transcript expression level from the upstream region of the TSS was two times lower than that from the downstream region (Condition 2; Middle panel). The remaining genes (Right panel). The values in Figure 2C were normalized by the number of genes with pancRNAs having the top100 ranked RPKM. **(D)** The genes with the top (left) and the bottom (right) 100 ranked sense RPKMs in the downstream region of their TSSs, respectively.

Our previous studies demonstrated that antisense transcripts from promoter regions could activate the sense transcription of the same locus [23,24]. Hence, we analyzed HtH transcript pairs, rather than overlapping transcription. In order to examine the switching-point of the bi-transcriptional direction, we focused on the genomic regions around TSSs of the reference genes. First, we adjusted the TSS of each reference gene according to the mapped reads of each tissue sample (see Methods). This adjustment is important for determining the precise distribution of mapped reads around TSSs. In fact, the ENCODE project showed that approximately 48% of the CAGE-identified TSSs are located hundreds of base pairs away from annotated GENCODE TSSs, indicating the requirement for this adjustment of TSSs [25]. Then, we examined the distribution of sense and antisense mapped reads around the TSS of each mouse protein-coding gene (Figure 2A and Additional file 2: Figure S2, S4A, E). In order to focus on the ncRNA-expressing promoters, we removed the HtH-type promoters driving protein-coding gene expression in both directions from our datasets. We examined the longest open reading frame (ORF) in each region between +1 and +1,000 bp and those between −1000 and −1 bp relative to the TSS, respectively. The mean length of the longest ORFs in the upstream and downstream regions is 191.5 and 319.6, respectively, in the mouse dataset. In the chimpanzee dataset, the mean length of the longest ORFs in the upstream and downstream regions is 213.0 and 305.0, respectively. Next, we examined the distribution of the longest ORF size in the mouse and chimpanzee dataset (Additional file 2: Figure S5A, B). There is one peak around 200 nt for the upstream region. On the other hand, there are two peaks around 200 and 900 nt for the downstream region. The 900-nt-peak seemed to reflect the fraction consisting of protein-coding genes. Moreover, we examined whether regions between −1,000 and −1 bp relative to the TSS contained any conserved protein domains by using NCBI’s Conserved Domain Database [26]. Only 1.9% and 4.8% of all regions from −1 to −1,000 bp relative to the TSS contain any conserved protein domains in the mouse and chimpanzee dataset, respectively. In contrast, 20.3% and 15.5% of all regions from +1 to +1,000 bp relative to the TSS contain conserved protein domains in the mouse and chimpanzee dataset, respectively. These results suggest that the vast majority of the upstream regions in our datasets produced ncRNAs, although we cannot completely exclude the possibility that a fraction of these antisense transcripts encode very short proteins.

In order to investigate if antisense transcription occurs in conjunction with transcription of the corresponding mRNA, we examined the distribution of sense and antisense mapped reads around the TSSs of mouse genes. Toward this end, we selected the 100 genes with the most-highly expressed pancRNAs and the 100 genes with the most-weakly expressed pancRNAs, as indicated by RPKM (Figure 2B and Additional file 2: Figure S4B, F). In this selection, we did not consider mRNA expression level for the selection of genes. For the RPKM calculation of the pancRNAs, only antisense mapped reads in the upstream region of protein-coding genes were counted. For estimation of the promoter activity of protein-coding genes, we focused on the region between +1 and +1,000 bp relative to the TSSs. Both in the mouse and chimpanzee samples, more sense reads were mapped to the protein-coding genes with pancRNAs showing the top 100 ranked RPKM than to those with pancRNAs showing the bottom 100 ranked RPKM (p < 0.001; Additional file 2: Figure S4J, N, S6A, Table 2, and Additional file 1: Table S4). When we calculated RPKM of the protein-coding genes based on the reference gene structure, we again found that the protein-coding genes with pancRNAs showing the top 100 ranked RPKM were more highly expressed than those with pancRNAs showing the bottom 100 ranked RPKM (p < 0.001; Additional file 2: Figure S6B). From the 100 regions with the most-highly expressed pancRNAs, we extracted three types of genomic regions: 1) the expression level of antisense transcript from the upstream region of the TSS is at least five times higher than that from the downstream region, 2) the expression level of antisense transcript from the upstream region of the TSS is at least two times lower than that from the downstream region, and 3) the remaining regions not meeting condition 1) or 2). Then, we examined the distribution of sense and antisense mapped reads in each subgroup (Figure 2C and Additional file 2: Figure S4C, G). Although we cannot rule out a possible short association between ncRNA and the corresponding mRNA at their 5’-ends, RT-PCR detection of transcripts derived from pancRNA-bearing gene loci *Pacsin1* and *Kcnmb4* supported the positive correlation between pancRNA and mRNA expression (Figure 1). We investigated two representative genes and confirmed that pancRNA and mRNA transcribed from the HtH promoter regions did not overlap with each other, which is in consistent with our directional RNA-seq data (Figure 1C, D). Therefore, it seemed likely that single-stranded ncRNAs function to activate the expression of the corresponding mRNAs via a mechanism independent of RNA-RNA duplex formation.

**Table 2:** RPKM of the upstream and downstream regions of TSSs of genes belonging to each subgroup

**Cerebral cortex**	**Upstream region**		**Downstream region**	
	**Antisense RPKM**	**Sense RPKM**	**Antisense RPKM**	**Sense RPKM**
Total genes	15.1	10.7	4.9	145.0
Top 100 ranked antisense RPKM located upstream^a^	734.3	9.1	195.6	267.5
& Low antisense RPKM located downstream^b^	427.5	5.9	22.8	181.5
& Middle antisense RPKM located downstream^b^	165.0	2.2	56.4	74.9
& High antisense RPKM located downstream^b^	141.8	1.0	116.4	11.1
Bottom 100 ranked antisense RPKM located upstream^c^	0.0	9.0	0.4	88.0
Top 100 ranked sense RPKM located downstream^d^	26.4	278.4	7.3	2054.4
Bottom 100 ranked sense RPKM located downstream^e^	3.3	0.3	2.9	0.0

The values in this table are normalized by the number of genes.

^a^The genes with the top 100 ranked antisense RPKM in the upstream region of their TSSs.

^b^The values are divided into three groups. The genes whose antisense transcirpt expression level from the upstream region of the TSS was five times higher than that from the downstream region (Condition 1; Low antisense RPKM in downstream region). The genes whose antisense transcript expression level from the upstream region of the TSS was two times lower than that from the downstream region (Condition 2; High antisense RPKM in downstream region). The remaining genes (Middle antisense RPKM in downstream region).

^c^The genes with the bottom 100 ranked antisense RPKMs in the upstream region of their TSSs.

^d^The genes with the top 100 ranked sense RPKMs in the downstream region of their TSSs.

^e^The genes with the bottom 100 ranked antisense RPKMs in the downstream region of their TSSs.

When we selected the genes with the top and the bottom 100 ranked RPKM in the downstream region of their TSSs, pancRNAs were not always associated with these genes (Figure 2D, Table 2, Additional file 2: Figure S4D, H, L, P and Additional file 1: Table S4). It is likely that constitutively expressed genes are generally not associated with pancRNA. These data suggested that highly expressed pancRNAs transcribed from the upstream regions of TSSs tended to be associated with the expression of the corresponding mRNAs in a coordinated manner, but highly expressed mRNAs were not always associated with the expression of pancRNAs.

### The tissue-specific expression of pancRNAs showed a positive correlation with that of the corresponding mRNAs

**Figure 3:**
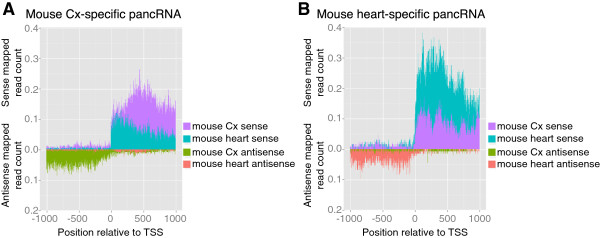
**Expression of pancRNAs was accompanied by that of corresponding mRNAs in a tissue-specific manner.** The distribution of sense and antisense mapped reads derived from the cerebral cortex and heart samples around TSSs of **(A)** the cerebral cortex- and **(B)** heart-specific pancRNA-bearing genes, respectively. In this analysis, we defined a pancRNA whose RPKM was higher than 0.3 in one tissue and lower than 0.1 in the other as a tissue-specific pancRNA. The values in this figure were normalized by the number of genes.

In the light of above observations, we thought it possible that pancRNAs could activate the transcription of the corresponding mRNAs in a tissue-specific manner. To test this possibility, we identified tissue-specific pancRNA-bearing genes based on the RPKM value of the candidate pancRNAs starting upstream of the TSSs. We examined the distribution of sense and antisense mapped reads derived from the mouse cerebral cortex and heart samples around the TSSs of the cerebral cortex- or heart-specific genes, respectively (Figure 3A, B and Table 3). The results showed that, for instance, for cerebral cortex-specific pancRNA-bearing genes, more sense reads corresponding to mRNAs derived from the cerebral cortex sample were mapped to the downstream of the TSS than such sense reads those derived from the heart sample. The same held true for heart-specific pancRNA-bearing genes. Therefore, we concluded that the expression of pancRNA was associated with the preferential upregulation of the corresponding mRNA in a given tissue. Basal mRNA expression was detected to some extent without pancRNA expression in the heart samples, and increased expression of the corresponding pancRNA was associated with higher gene expression in the cerebral cortex, suggesting that the expression of pancRNAs could enhance the corresponding mRNA expression rather than triggering it. Information on the comparison between cerebellum and heart is shown in Additional file 2: Figure S7 and Additional file 1: Table S5, and indicates a similar tendency of a positive correlation between pancRNA and mRNA expressions.

**Table 3:** RPKM of the upstream and downstream regions of TSSs of genes with tissue-specific pancRNAs

**Cerebral cortex vs Heart**		**Upstream region**		**Downstream region**	
		**Antisense RPKM**	**Sense RPKM**	**Antisense RPKM**	**Sense RPKM**
Cerebral cortex-specific pancRNA-bearing genes	Cerebral cortex	98.1	18.5	12.9	369.9
	Heart	2.2	4.6	2.9	163.8
Heart-specific pancRNA-bearing genes	Cerebral cortex	3.7	12.1	1.4	172.3
	Heart	83.7	18.6	6.4	516.6

The values in this table were normalized by the number of genes. In this analysis, we defined a pancRNA whose RPKM was higher than 0.3 in one tissue and lower than 0.1 in the other as a tissue-specific pancRNA.

**Figure 4:**
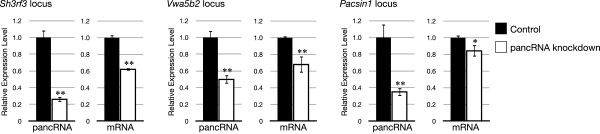
**Knockdown of pancRNAs could decrease the expression level of the corresponding mRNAs.** The effects of each pancRNA knockdown on expression level of *Sh3rf3*, *Vwa5b2* and *Pacsin1* in mouse neurons. In each experiment, the shRNA against the pancRNA corresponding to the examined gene was used. Expression levels determined by real-time PCR are the mean ± SEM (n = 3) relative to that for mRNA or pancRNA in empty vector-transfected neurons. **p < 0.01 and *p < 0.05; Student’s t test.

In order to support our hypothesis, we examined the function of three representative pancRNA-bearing genes selected from the mouse cerebral cortex-specific pancRNA-bearing genes, *Sh3rf3*, *Vwa5b2* and *Pacsin1*. We performed quantitative RT-PCR to detect the expression level of pancRNA and the corresponding mRNA in the mouse cortical neurons after pancRNA knockdown as described in Methods (Figure 4). As expected, knockdown of each pancRNA (*pancSh3rf3*, *pancVwa5b2* and *pancPacsin1*) significantly decreased the expression of the corresponding mRNA. These results suggest that pancRNAs could enhance the corresponding mRNA expression. Furthermore, to show a direct link between the expression level of pancRNAs and their corresponding mRNAs in several cell types, we calculated sense and antisense RPKM in the downstream and upstream regions of the TSSs, respectively, and examined the Pearson correlation coefficient between the sense and antisense RPKM at these three gene loci in various tissues. For this analysis, we utilized processed data on mouse directional RNA-seq of 19 different tissues and primary cells available from NCBI Gene Expression Omnibus (GSE29278) [27,28]. The average of these three correlation coefficients was 0.86 (Additional file 1: Table S6). Taken together, our results indicate that a fraction of pancRNAs are expressed from the HtH regions and support the notion that bidirectional promoter regions function in *cis* to regulate gene expression via pancRNA production for setting up precise tissue-specific gene expression profiles.

### Sequence characteristics of pancRNA-bearing genes

We hypothesized that the presence or absence of pancRNA was attributable to the genomic DNA information. To test this, first we used the Gardiner-Garden-Frommer based CGIs available from the UCSC table browser [29]. Notably, 92.3% of the candidate pancRNA-bearing genes overlapped with CGIs in the mouse (Table 4). A bias for CGIs was also found in chimpanzee samples (Additional file 1: Table S7). These results showed that the bidirectional promoter regions of protein-coding genes exhibited a strong bias for CGIs, supporting the presence of genomic characteristics of pancRNA-bearing gene promoter regions.

**Table 4:** The bias of the pancRNA-bearing protein-coding genes for CpG islands in various mouse tissues

	**Candidate pancRNA-bearing genes**
	**With CpG islands in their promoter regions**^**a**^
Cerebral cortex	92.8%
Cerebellum	91.4%
Heart	92.3%

^a^The percentage of pancRNA-bearing protein-coding genes harboring CpG islands in their promoter regions.

**Figure 5:**
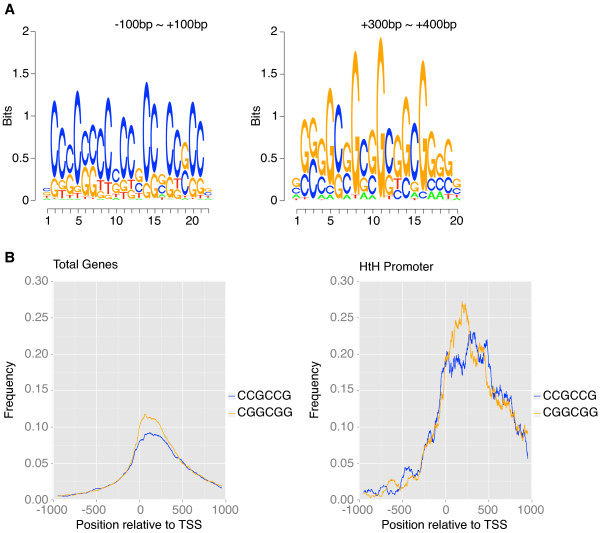
**Sequence characteristics of pancRNA-bearing genes in the mouse cerebral cortex. (A)** The sequence logos found in the regions from −100 bp to +100 bp and from +300 bp to +400 bp relative to the TSS of candidate pancRNA-bearing genes. **(B)** The observed frequencies of the “CCGCCG” and “CGGCGG” sequences across the regions around the TSSs of all promoter regions (left) and of candidate pancRNA-bearing genes’ promoter regions (right).

Next we considered the possibility that a fraction of CGIs may have signature sequences that direct pancRNA expression. Using the dataset of candidate pancRNA-bearing genes, we performed *de novo* motif discovery. We found that in all of the mouse tissue samples examined, several CCG repeats were located between −100 and +100 bp (p < 0.0002; Figure 5A and Additional file 2: Figure S8A, C). Moreover, we found that in all of these tissues, several CGG repeats, complementary to the CCG repeats, were located in the downstream region starting from +100 bp. CCG and CGG repeats were overrepresented at similar genomic locations in chimpanzee samples (p < 0.0002; Additional file 2: Figure S8E, G).

The average repeat numbers of CCG and CGG were 2.14 and 2.16, and the maximum repeat numbers of CCG and CGG were 15 and 11, respectively. Then, to examine whether the distribution of CCG and CGG repeats was preferentially observed in the promoter regions of candidate pancRNA-bearing genes, we calculated the observed frequency of “CCGCCG” and “CGGCGG” sequences across the regions around the TSSs (Figure 5B and Additional file 2: Figure S8B, D, F, H). Like the observed consensus repeat sequences, both “CCGCCG” and “CGGCGG” sequences were significantly enriched in the promoter regions of candidate pancRNA-bearing genes, reflecting the high rate of overlap of the promoter regions of candidate pancRNA-bearing genes with CGIs.

Analysis using all promoter sequences also showed that the peaks of the distributions of these two six-base sequences occurred at a similar position relative to the TSSs. In contrast, analysis using the promoter sequences of candidate pancRNA-bearing genes showed that the peaks were located at different positions. To examine how many promoter regions harbor both of these repeat sequences, we calculated the percentage of the promoter regions with “CCGCCG” and “CGGCGG” sequences (Table 5 and Additional file 1: Table S8). We found that 47.8% of candidate pancRNA-bearing genes in the cerebral cortex harbored both sequences, whereas only 19.7% of all promoter regions did.

**Table 5:** The percentage of genes expressed in various mouse tissues with both “CCGCCG” and “CGGCGG” sequences

**All genes**	**Candidate pancRNA-bearing genes**		
	**In cerebral cortex**	**In cerebellum**	**In heart**
19.7%	47.8%	47.3%	47.5%

Taken together, these results showed that bidirectional transcription occurred frequently in promoter regions, and that such bidirectional promoter regions exhibited a bias toward GC-rich sequences, especially CCG and CGG repeats, potentially affecting the transcription of protein-coding genes with tissue-dependent expression.

## Discussion

### Assessment of directional RNA-seq data

In our directional RNA-seq analyses, the average percentages of uniquely and multiply mapped reads in the valid reads determined by using TopHat were 81.4% and 94.7% for the mouse, and 80.1% and 87.8% for the chimpanzee, respectively (Additional file 1: Table S1). These mapping rates are compatible with those of RNA sequencing analysis in the ENCODE project, in which the average percentage of all mapped reads-pairs corresponding to total reads in polyA+ RNA sequencing is 88.7% [25]. In our analysis, we used the human instead of the chimpanzee genome as a reference. On average, 72.4% and 78.2% of the valid reads in directional RNA-seq data of the chimpanzee cerebral cortex were uniquely mapped onto the chimpanzee and the human genome sequence, respectively, except for random chromosome sequences (Additional file 1: Table S2), validating the sequence similarity between the chimpanzee and the human genome. In fact, the number of transcripts with protein-coding gene biotype is much smaller in the chimpanzee database than in the human database. 19,895 transcripts have been registered in the Chimpanzee Ensembl genes with protein-coding gene biotypes, whereas 197,870 transcripts have been registered in the Human Ensembl genes with protein-coding gene biotypes. Therefore, the massive annotation of the human transcripts enabled us to determine the transcription characteristics around the TSSs of the protein-coding genes in the chimpanzee, as shown in Additional file 2: Figure S4I, M.

The valid reads in our directional RNA-seq data of the mouse cerebral cortex samples covered 25.0% of the mouse genome (Table 1). Those of the chimpanzee cerebral cortex samples covered 24.5% of the human genome (Additional file 1: Table S3). Comparable numbers were found by the ENCODE project, showing that the average nucleotide coverage of the human genome in several cell types is about 20% [25]. Therefore, taken together with the mapping quality, the rich reference data, and the reproducibility of our data, the sequencing depth of our datasets was high enough for comprehensive detection of the transcribed regions in the genome, as discussed below.

### The importance of single-stranded ncRNAs for gene regulations

The capacity of a ncRNA to form a duplex with partially complementary RNA is one of the criteria for classifying the properties of ncRNAs. Previous studies have indicated that the formation of RNA-RNA duplexes is important for post-transcriptional regulation [6-10,13]. Theoretically, ncRNA-mRNA duplex formation could decrease the gene expression level through both pre- and post-transcriptional regulation [11,12]. In contrast, single-stranded ncRNAs generally regulate transcription in cooperation with chromatin modification factors (see, for example, [19]), although their underlying mechanisms are largely unknown. Our analyses suggested that pairs of sense and antisense transcripts expressed in a tissue were rather rare. Only 0.7%, 1.3% and 0.7% of the genome was bidirectionally transcribed in the mouse cerebral cortex, cerebellum and heart, respectively (Table 1). Even in the bidirectionally transcribed regions, either the top or bottom strand was preferentially utilized (Additional file 2: Figure S3). These features of the transcriptional landscape were seen in the chimpanzee tissue samples as well (Additional file 1: Table S3 and Additional file 2: Figure S3). Thus, in mammals, there are a non-negligible number of single-stranded ncRNAs that can function in a cell.

### The transcriptional activation mediated by pancRNAs in the bidirectional promoter regions

Among single-stranded ncRNAs, we focus on the ncRNAs transcribed from mRNA promoter regions. In this study using mouse and chimpanzee tissue samples, we showed that antisense ncRNAs transcribed from promoter regions could be classified into two categories according to the location of their TSSs relative to those of the corresponding mRNAs (Figure 2C and Additional file 2: Figure S4C, G, K, O). The first category is composed of antisense ncRNAs overlapping with the corresponding mRNAs. These antisense ncRNAs can downregulate the corresponding mRNAs, because such antisense ncRNAs are known to downregulate the mRNAs via the formation of ncRNA-mRNA duplexes [7,10-12]. For example, the expression level of the transcription factor PU.1 is downregulated by the antisense ncRNA complementary to the mRNA via the formation of an mRNA-ncRNA duplex [11]. The other category is composed of antisense ncRNAs starting from regions upstream of the TSSs of the corresponding mRNAs, i.e., pancRNAs. Here, we showed a positive correlation between the expression of pancRNAs and the corresponding mRNAs, which is consistent with reports of transcriptional activation mediated by the overexpression of pancRNAs that do not hybridize with the corresponding mRNAs [23,24]. Overexpression of *Khps1*, one such pancRNA transcribed from the *Sphk1* gene region, causes demethylation of CpG sites in the promoter region of *Sphk1* to activate the transcription of *Sphk1*[23]. In this study, we found that the expression of hundreds of pancRNAs was accompanied by the expression of the corresponding mRNAs in a tissue-specific manner (Figure 3 and Additional file 2: Figure S7). Moreover, we showed that knockdown of cerebral cortex-specific pancRNA significantly decreased the expression of the corresponding mRNA in mouse neurons (Figure 4). Therefore, we propose that, like some pancRNAs that we have previously identified [23,24], pancRNAs could act as tissue-specific transcriptional facilitator of the expression of the corresponding mRNAs via epigenetic mechanisms. Recent data from the ENCODE project are in accord with this idea: many lncRNAs show a tissue-specific expression pattern that is positively correlated with that of the mRNAs with which they share a single bidirectional promoter [25,30]. Positive correlation between the expression of pancRNAs and corresponding mRNAs was seen in both the mouse and chimpanzee tissue samples (Figure 2 and Additional file 2: Figure S4), raising the possibility that the mode of regulation of mRNA expression by pancRNA is similar between mouse and chimpanzee.

We now think that many pancRNAs exist and enhance mRNA expression at the genome-wide level, but not all mRNAs are under pancRNA regulation, because the highly expressed mRNAs were not associated with corresponding antisense ncRNAs (Figure 2D). This result also suggests that expression of mRNAs may not enhance the corresponding pancRNA expression. Based on our data, lncRNAs that function in setting up the chromatin structure can be subgrouped into at least three categories: 1) those containing RNA domains that specifically interact with chromatin modifiers to modulate thousands of loci (eg. *HOTAIR*), 2) those functioning together with complementary RNAs (eg. SAT), and 3) those that act in *cis* to specifically set up active chromatin status in a sequence-specific manner (pancRNA).

### Abundant CCG and CGG repeats and CGIs as hallmarks of pancRNA-mediated gene regulation

In our genome-wide analysis, we examined the sequence characteristics important for the transcription of pancRNAs. We found that more than 90% of candidate pancRNA-bearing genes overlapped with CGIs, and that CCG and CGG repeats appeared frequently around TSSs of such genes in the mouse and chimpanzee tissue samples (Table 4, Figure 5, Additional file 1: Table S7 and Additional file 2: Figure S8). Since at least some pancRNAs can induce DNA demethylation of CpG sites in promoter regions [23,24], there must be CpG sites in the promoter regions of such pancRNA-bearing genes. Therefore, it is logical to assume that pancRNAs are preferentially derived from regulatory GC-rich sequences. Notably, not all CGI promoter regions show bidirectional promoter activity. Since we examined only three tissues, cerebral cortex, cerebellum and heart, in this study, it will be important to verify that CGI promoters in other tissues also act as pancRNA-bearing gene promoters.

It is possible that there are some additional sequence characteristics important for the bidirectional transcription from pancRNA-bearing promoter regions. It is interesting to note that there is a gap between the peak of the CCG and the CGG repeats (Figure 5 and Additional file 2: Figure S8). We consider that this gap probably plays a key role in the transcription of pancRNAs. Intriguingly, the regions with these characteristics are included in the GC-skewed regions where the distributions of guanines and cytosines are biased. It was reported that transcription through GC-skewed regions led to the formation of DNA-DNA-RNA triple helix structures, termed R loop structures. R loop formation has been shown to protect against DNA methylation [31]. Therefore, we consider that GC skew results from the biased distribution of CCG and CGG repeats around TSSs, and that a fraction of pancRNAs may be involved in the formation of the DNA-DNA-RNA triple helix structures during the process of DNA demethylation. However, not all pancRNA-bearing promoter regions contain both “CCGCCG” and “CGGCGG” sequences (Table 5 and Additional file 1: Table S8). One possible explanation for this fact is that there may be as yet unknown physical features of the DNA sequences regarding how the distribution of guanines and cytosines affects strand asymmetry formation. Although the mechanisms of DNA demethylation induced by pancRNA are currently unknown, identification of these two consensus repeats may provide a clue to unravel how pancRNAs mediate transcriptional activation.

## Conclusions

In conclusion, we show here that a significant number of single-stranded ncRNAs and pancRNAs exist in a cell. Our findings suggest that, in mammals, specific DNA sequences regulate the expression of pancRNAs, which enhance the expression level of the corresponding mRNAs in a tissue-specific manner. The sequences enriched in pancRNA-bearing genes, CCG and CGG repeats, may be important for the expression of pancRNAs.

## Methods

### Tissue preparations

C57BL6 mice (*Mus musculus*; Japan SLC) were kept under a lighting regime of 14 h illumination and 10 h darkness (lights on between 05:00 and 19:00 h) and were allowed free access to food and water. Tissue samples from mice (16 weeks of age; male) were collected and immediately frozen in liquid nitrogen and stored at −80°C until use. Thanks to the Great Ape Information Network (GAIN) and Kumamoto Sanctuary, Wildlife Research Center, Kyoto University, the BA10 area and cerebellum were collected from a chimpanzee (*Pan troglodytes*; about 28-year-old female). The total RNAs were isolated from the mouse cerebral cortex, cerebellum and heart, and the chimpanzee cerebral cortex and cerebellum. This study was approved by the Animal Research Committee, Kyoto University, Japan, and these experimental procedures were conducted according to the Regulation on Animal Experimentation at Kyoto University.

### shRNA knockdown of pancRNA in primary murine cortical neurons

shRNA oligos were annealed and ligated into pLLX-shRNA vector, which also carries a GFP marker. All oligo sequences are described in Additional file 1: Table S10. Human embryonic kidney cells were used as producers of lentiviruses that contained pLLX-shRNA vectors. Viral preparations were applied at 1 day *in vitro* (DIV).

Neurons were isolated from the cerebral cortex of ICR mouse embryos at embryonic day 17.5. After removal of meninges, cerebral cortices were dissected and collected in ice-cold Ca^2+^- and Mg^2+^-free HBSS (Hank’s balanced salt solution; SIGMA) with 4.17 mM NaHCO_3_. These cortices were incubated in Ca^2+^- and Mg^2+^-free PBS containing 0.33 g/l L-cysteine (nacalai tesque), 0.33 g/l BSA (SIGMA), 8.33 g/l glucose, 500 U/ml DNase I (SIGMA) and 3.3% papain from papaya latex (SIGMA) for 20 min at 37°C and washed with MEM Alpha (gibco) supplemented with 0.6% glucose, 5% FBS, 1% Antibiotic-Antimycotic Mixed Stock Solution (Nacalai Tesque) followed by trituration in HBSS containing 3.0 g/l MgSO_4_7H_2_O and 500 U/ml (Sigma). After two washes with MEM Alpha, cells were seeded on poly-L-lysine-coated dish and incubated in MEM Alpha. After incubation for 3 hours, the medium was replaced with Neurobasal medium (Gibco) containing 2% B27 supplement (Invitrogen), 1% GlutaMax (Invitrogen) and 1% Antibiotic-Antimycotic Mixed Stock Solution in a humidified atmosphere (5% CO_2_/95% air) at 37°C. Viral preparations were applied at 1 DIV. After 3 DIV, half of the culture medium was replaced with fresh medium supplemented with 10 μM AraC (SIGMA). All experiments were performed using 5 DIV cultures.

### Directional RNA sequencing

Directional RNA-seq samples were prepared according to a slight modification of the protocol provided by Illumina. Briefly, cDNA libraries were prepared starting from 5 μg of total RNA as follows. First, total RNA was selected twice with Sera-Mag Magnetic Oligo (dT) Beads (Thermo Scientific) to isolate polyA+ RNA. The fraction of rRNA was found to be less than 2% in each polyA+ RNA sample by using a Total RNA Pico Bioanalyzer chip (Agilent). polyA+ RNA was fragmented by heating at 94°C for 3 min in 1 × fragmentation buffer (Affymetrix), followed by ethanol precipitation. Fragmented RNA was decapped with TAP, followed by extraction with PCI and ethanol precipitation. Fragmented and decapped RNA was 3′-dephosphorylated using Antarctic phosphatase (NEB). The RNA was 5′-phosphorylated using T4 polynucleotide kinase (NEB). The modified RNA was cleaned up with an RNeasy MinElute kit (Qiagen). The RNA was ligated to 1 × v1.5 sRNA 3′ adaptor (Illumina) with T4 RNA ligase 2, truncated K277Q (NEB) at 4°C overnight. This RNA was ligated to SRA 5′ adaptor (Illumina) with T4 RNA ligase (Illumina) at 20°C for 1 hr. cDNA was synthesized with specific RT primer and the SuperScriptIII First-Strand Synthesis System (Life Technologies Co.). Before the amplification, two cDNA replicates were prepared. Each cDNA was amplified with Phusion DNA Polymerase (Finnzymes), independently. Thermal-cycling conditions were as follows: 30 sec at 98°C, 12 cycles of 98°C for 10 sec, 60°C for 30 sec, and 72°C for 15 sec, followed by 10 min at 72°C. The PCR product was purified twice with AMPure XP (Beckman Coulter) to generate a library and analyzed on a DNA1000 Bioanalyzer chip (Agilent) for precise quantification of molarity. After confirmation of the high quality of the cDNA library samples, we sequenced the mouse cerebral cortex and the chimpanzee cerebral cortex and cerebellum for 101 bp single-end reads (first run) and the mouse cerebellum and heart for 51 bp single-end reads (second run) using two lanes of the Illumina HiSeq 2000 per sample with the small RNA sequencing primer (Illumina) according to the manufacturer’s instructions.

### Data mining

To trim adaptor sequences, the reads were clipped from the 3'-end of reads. Then, the nucleotides were eliminated from the 3‘-end of reads to the nucleotides whose sequencing accuracy was lower than 99%. Finally, the reads longer than 50 nt were trimmed from the 3’-end of reads to 50 nt and the reads shorter than 20 nt were removed.

We mapped sequencing reads from each sample onto the respective mouse or human reference genome sequences (mm9 or hg19) except for random chromosome sequences using TopHat (v.2.0.4) under the default parameters [32]. We mapped the sequencing reads of chimpanzee samples to the human reference genome (hg19) because the human genome shows very high similarity with the chimpanzee genome, and the annotation of the human genome is better organized, as explained in the Discussion section. After this mapping process, we gathered multiple reads into one read based on the mapping information of the 5’-end of each read in order to remove sequence duplication.

In this analysis, we defined the regions that contained origins for both sense and antisense transcripts as bidirectionally transcribed regions. Based on our transcript mapping and reference gene information, we adjusted the genomic locations of TSSs. For this purpose, we identified the transcript-enriched regions using MACS software under the non-MACS model (−−nomodel; v.1.4.1)[33]. In this analysis, minimum FDR cutoff for peak detection is 0.05. We defined the 5’-ends of these regions as the adjusted TSS when the 5’-ends were located from −1,000 bp to +1,000 bp from the reference TSS. In drawing the distribution of sense and antisense mapped reads around TSSs, we eliminated the furthest-upstream reads composing the transcript-enriched regions because these reads were utilized for the adjustment of the genomic location of TSSs that resulted in the intentional overrepresentation of read enrichment at the TSSs.

For the efficient calculation of density of the ratio of sense mapped reads to antisense mapped reads, genomic locations every 100 bp were first extracted, and then filtered based on the criteria that more than one sense mapped read and antisense mapped read existed in order to pick up the genomic fragments in which both strands were actively utilized. The 100-bp fragments starting from the representative genomic locations were further screened based on the criterion that the total length of the sense or antisense mapped reads was more than 300 bp.

### RT-PCR analysis

To examine RNA expression, total RNA isolated from tissues with TRIzol reagent (Life Technologies Co.) was treated with DNase I (Life Technologies Co.) and reverse-transcribed with each respective gene-specific primer or oligo-dT primer using the SuperScriptIII First-Strand Synthesis System (Life Technologies Co.). Strand-specific PCR was carried out with specific primers for each transcript (see Additional file 1: Table S9).

Quantitative PCR was performed with KAPA SYBR Fast qPCR Kit (KAPA Biosystems) using an Applied Biosystems StepOnePlus Real-Time PCR System (Life Technologies Co.). Quantitative PCR was carried out with specific primers for each transcript (see Additional file 1: Table S11). Relative quantities of mRNA were normalized by the glyceraldehyde-3-phosphate dehydrogenase (*Gapdh*) mRNA content.

### RNA quantification and the identification of candidates for bidirectional promoter regions

To quantify the transcription level around TSSs based on directional RNA-seq data, we counted the number of mapped reads in the region from −1,000 bp to +1,000 bp relative to the TSS and normalized this number by RPKM. In order to focus on the promoter regions where ncRNA is transcribed, we removed the promoter regions where two mRNAs were transcribed in a HtH manner from promoters of protein-coding genes with Ensembl Gene ID (NCBIM37 for the mouse genome and GRCh37 for the human genome) available from Ensembl Genes Database [34]. Cufflinks (v2.1.1) was used with default parameters for quantification of RPKM of known protein-coding genes [35].

We utilized the MACS output file, which contains the location of transcript-enriched regions. We sorted the transcript-enriched regions according to the genomic location. If a minus transcript was located next to a plus transcript, we regarded their boundary sequence as the target for further analysis of the genomic properties driving bidirectional transcription. We narrowed down the candidates based on the minus-plus pitch. We only analyzed the minus-plus pitches that were mutually located within 2,000 bp.

### Calculation of ORF length

To examine the length of ORFs, we used EMBOSS:getorf [36] with the sequences of the downstream region (+1 to +1,000 bp) and of the upstream region (−1,000 to −1 bp) of the candidate pancRNA-bearing genes. In this analysis, we defined an ORF as a region which begins with a START codon and ends with a STOP codon.

### *De novo* motif discovery

For motif discovery, we employed the rGADEM package (v.1.0.1) [37], which is available through Bioconductor [38], with the default parameter (P-value < 0.0002) [39] with DNA sequences from −1,000 bp to +1,000 bp relative to TSSs of the candidate pancRNA-bearing genes. We calculated the observed frequencies of “CCGCCG” or “CGGCGG” sequences from −1,000 bp to +1,000 bp relative to TSSs of the candidate pancRNA-bearing genes with sliding window of width 100 bp. The average numbers of “CCGCCG” and “CGGCGG” found in a sequence were plotted with a sliding window of width 100 bp.

## Abbreviations

ncRNA: Non-coding RNA; TSS: Transcription start site; pancRNA: Promoter-associated ncRNA; SAT: Sense-antisense transcript; lncRNA: Long intergenic non-coding RNA; CGI: CpG island; HtH: Head-to-head; RPKM: Reads per kilobase per million mapped reads; ORF: Open reading frame.

## Competing interests

The authors declare that they have no competing interests.

## Authors’ contributions

MU conceived the project, designed and performed experiments, conducted bioinformatic analysis and drafted the manuscript. ON conducted bioinformatic analysis. YG contributed to the sample preparation for directional RNA-seq. KN designed experiments and drafted the manuscript. KA designed experiments and drafted the manuscript. TI conceived the project, designed experiments, conducted bioinformatic analysis, coordinated the study and drafted the manuscript. All authors read and approved the final manuscript.

## Supplementary Material

Additional file 1 **Table S1.** Summary of directional RNA sequencing. **Table S2.** Comparison of the number of the uniquely mapped reads in directional RNAseq data of chimpanzee samples mapped onto the human versus the chimpanzee genome. **Table S3.** The percentage of transcribed regions in the whole genome in various chimpanzee tissues. **Table S4.** RPKM of the upstream and downstream regions of TSSs of genes belonging to each subgroup in each indicated tissue. **Table S5.** RPKM of the upstream and downstream regions of TSSs of genes with tissuespecific pancRNAs in various tissues. **Table S6.** Pearson correlation coefficient between the expression level of pancRNA and the corresponding mRNA. **Table S7.** The bias of the pancRNA-bearing protein-coding genes for CpG islands in various chimpanzee tissues. **Table S8.** The percentage of genes expressed in various chimpanzee tissues with both “CCGCCG” and “CGGCGG” sequences. **Table S9.** Primers for strand-specific RT-PCR analysis. **Table S10.** shRNA sequences. **Table S11.** Primers for quantitative RT-PCR analysis. Click here for file

Additional file 2 **Figure S1.** The depth of coverage of each base in the mouse genome with reads from directional RNAseq data of the mouse cerebral cortex. **Figure S2.** Either top or bottom strand of genome is preferentially utilized for transcription. **Figure S3.** Density plots of the ratio of top strand-mapped reads to bottom strand-mapped reads. **Figure S4.** The distribution of sense and antisense mapped reads around the TSS of each gene fraction in the mouse cerebellum, mouse heart, chimpanzee cerebral cortex, and chimpanzee cerebellum. **Figure S5.** The distribution of longest ORFs in downstream region and upstream region. **Figure S6.** The Average RPKM of genes bearing pancRNAs with the 100 top-ranked RPKM and those with the bottom-ranked RPKM relative to RPKM of total genes in all samples. **Figure S7.** Expression of pancRNAs was accompanied by that of corresponding mRNAs in a tissue-specific manner. **Figure S8.** Sequence characteristics of pancRNA-bearing genes in the mouse cerebellum, mouse heart, chimpanzee cerebral cortex and chimpanzee cerebellum. Click here for file
