# Supplementary material for: Bidirectional promoters are the major source of gene activation-associated non-coding RNAs in mammals
Source: BMC Genomics. 2014 Jan 17;15:35. doi: 10.1186/1471-2164-15-35 (PMC3898825; doi:10.1186/1471-2164-15-35)
Supplement: Additional file 1 — Table S1. Summary of directional RNA sequencing. Table S2. Comparison of the number of the uniquely mapped reads in directional RNAseq data of chimpanzee samples mapped onto the human versus the chimpanzee genome. Table S3. The percentage of transcribed regions in the whole genome in various chimpanzee tissues. Table S4. RPKM of the upstream and downstream regions of TSSs of genes belonging to each subgroup in each indicated tissue. Table S5. RPKM of the upstream and downstream regions of TSSs of genes with tissuespecific pancRNAs in various tissues. Table S6. Pearson correlation coefficient between the expression level of pancRNA and the corresponding mRNA. Table S7. The bias of the pancRNA-bearing protein-coding genes for CpG islands in various chimpanzee tissues. Table S8. The percentage of genes expressed in various chimpanzee tissues with both “CCGCCG” and “CGGCGG” sequences. Table S9. Primers for strand-specific RT-PCR analysis. Table S10. shRNA sequences. Table S11. Primers for quantitative RT-PCR analysis. [file 1471-2164-15-35-S1.pdf]

Supplementary Table S1

Summary of directional RNA sequencing

	Sample	Total # reads	# Valid reads[a]	(%)[b]	# Mapped reads	(%)[c]	# Uniquely mapped reads	(%)[d]	# Uniquely mapped reads after removal of duplication	Top strand-mapped reads / Bottom strand-mapped reads[e]
1st run	Chimpanzee Cerebral Cortex (n=1)	75,012,641	70,894,403	94.5%	61,404,334	86.6%	55,436,357	78.2%	18,894,958	1.03
	Chimpanzee Cerebral Cortex (n=2)	77,141,984	72,913,619	94.5%	63,089,059	86.5%	56,935,814	78.1%	19,057,212	1.03
	Chimpanzee Cerebellum (n=1)	76,342,191	72,237,582	94.6%	64,283,120	89.0%	59,273,070	82.1%	22,562,341	1.04
	Chimpanzee Cerebellum (n=2)	76,081,033	71,963,443	94.6%	63,973,178	88.9%	58,955,648	81.9%	22,396,483	1.04
	Mouse Cerebral Cortex (n=1)	76,557,221	72,322,648	94.5%	68,635,440	94.9%	62,286,305	86.1%	18,552,890	1.02
	Mouse Cerebral Cortex (n=2)	76,687,574	72,437,179	94.5%	68,665,190	94.8%	62,418,231	86.2%	19,814,914	1.02
2nd run	Mouse Cerebellum (n=1)	220,787,053	192,894,809	87.4%	181,763,145	94.2%	164,728,033	85.4%	30,097,533	1.03
	Mouse Cerebellum (n=2)	238,989,445	207,347,311	86.8%	195,402,638	94.2%	177,060,051	85.4%	30,571,226	1.03
	Mouse Heart (n=1)	233,051,369	201,311,951	86.4%	190,937,364	94.8%	146,153,387	72.6%	18,243,843	1.01
	Mouse Heart (n=2)	220,364,813	191,584,172	86.9%	181,795,500	94.9%	139,227,099	72.7%	17,875,501	1.01

[a]The number of reads passing the read trimming

[b]The percentage of valid reads in the total reads

[c]The percentage of mapped reads in the valid reads

[d]The percentage of uniquely mapped reads in the valid reads

[e]The ratio of top strand- to bottom strand-mapped reads after removal of duplication

Supplementary Table S2

Comparison of the number of the uniquely mapped reads in directional RNA-seq data of chimpanzee samples mapped onto the human versus the chimpanzee genome

Sample	Reference genome	# Mapped reads	(%)[a]	# Uniquely mapped reads	(%)[b]
Chimpanzee Cerebral Cortex	human	62,246,697	86.6%	56,186,086	78.1%
	chimpanzee	60,036,046	83.5%	52,078,154	72.4%
Chimpanzee Cerebellum	human	64,128,149	88.9%	59,114,359	82.0%
	chimpanzee	62,768,664	87.1%	57,441,695	79.7%

[a]The percentage of mapped reads in the valid reads

[b]The percentage of uniquely mapped reads in the valid reads

Supplementary Table S3

The percentage of transcribed regions in the whole genome in various chimpanzee tissues

	Transcribed regions	Unidirectionally transcribed regions[a]	Bidirectionally transcribed regions[b]
Cerebral cortex	24.5%	23.6%	0.9%
Cerebellum	23.4%	22.2%	1.2%

[a]Regions where either sense or antisense transcripts (but not both) originated.

[b]Regions where both sense and antisense transcripts originated.

Supplementary Table S4

RPKM of the upstream and downstream regions of TSSs of genes belonging to each subgroup in each indicated tissue

Sample	Subgroup	Upstream region		Downstream region	
		Antisense RPKM	Sense RPKM	Antisense RPKM	Sense RPKM
Mouse Cerebellum	Total genes	19.4	11.3	8.1	174.8
	Top 100 ranked antisense RPKM located upstream[a]	790.7	15.2	213.2	370.5
	& Low antisense RPKM located downstream[b]	463.0	7.4	32.3	206.1
	& Middle antisense RPKM located downstream[b]	190.4	7.3	65.6	124.6
	& High antisense RPKM located downstream[b]	137.3	0.6	115.3	39.8
	Bottom 100 ranked antisense RPKM located upstream[c]	0.0	8.4	0.5	88.1
	Top 100 ranked sense RPKM located downstream[d]	50.1	236.3	22.7	1736.3
	Bottom 100 ranked sense RPKM located downstream[e]	2.1	0.3	1.9	0.0
Mouse Heart	Total genes	15.7	10.5	6.5	211.2
	Top 100 ranked antisense RPKM located upstream[a]	848.6	19.4	207.2	489.1
	& Low antisense RPKM located downstream[b]	579.0	15.2	27.9	246.8
	& Middle antisense RPKM located downstream[b]	120.7	1.1	41.5	129.3
	& High antisense RPKM located downstream[b]	148.9	3.1	137.9	113.0
	Bottom 100 ranked antisense RPKM located upstream[c]	0.0	8.9	0.5	121.6
	Top 100 ranked sense RPKM located downstream[d]	70.4	181.6	40.9	2542.2
	Bottom 100 ranked sense RPKM located downstream[e]	2.0	0.3	1.6	0.0
Chimpanzee Cerebral Cortex	Total genes	16.2	10.0	5.9	114.3
	Top 100 ranked antisense RPKM located upstream[a]	653.6	7.3	150.0	231.0
	& Low antisense RPKM located downstream[b]	401.4	5.1	18.7	146.6
	& Middle antisense RPKM located downstream[b]	147.7	1.7	48.5	44.0
	& High antisense RPKM located downstream[b]	104.4	0.5	82.8	40.4
	Bottom 100 ranked antisense RPKM located upstream[c]	0.0	9.5	0.9	78.4
	Top 100 ranked sense RPKM located downstream[d]	25.3	94.9	14.5	1636.6
	Bottom 100 ranked sense RPKM located downstream[e]	5.4	1.0	5.1	0.0
Chimpanzee Cerebellum	Total genes	16.1	10.7	6.3	102.4
	Top 100 ranked antisense RPKM located upstream[a]	649.9	26.6	211.2	214.5
	& Low antisense RPKM located downstream[b]	402.1	24.2	27.9	131.2
	& Middle antisense RPKM located downstream[b]	85.0	0.7	24.7	22.8
	& High antisense RPKM located downstream[b]	162.8	1.6	158.6	60.4
	Bottom 100 ranked antisense RPKM located upstream[c]	0.0	9.3	0.5	65.1
	Top 100 ranked sense RPKM located downstream[d]	59.9	119.2	44.8	1484.9
	Bottom 100 ranked sense RPKM located downstream[e]	4.4	0.6	4.8	0.0

The values in this table are normalized by the number of genes.

[a]The genes with the top 100 ranked antisense RPKM in the upstream region of their TSSs

[b]The values are divided into three groups. The genes whose antisense transcript expression level from the upstream region of the TSS was five times higher than that from the downstream region (Condition 1; Low antisense RPKM in downstream region). The genes whose antisense transcript expression level from the upstream region of the TSS was two times lower than that from the downstream region (Condition 2; High antisense RPKM in downstream region). The remaining genes (Middle antisense RPKM in downstream region).

[c]The genes with the bottom 100 ranked antisense RPKMs in the upstream region of their TSSs

[d]The genes with the top 100 ranked sense RPKMs in the downstream region of their TSSs

[e]The genes with the bottom 100 ranked antisense RPKMs in the downstream region of their TSSs

Supplementary Table S5

RPKM of the upstream and downstream regions of TSSs of genes with tissue-specific pancRNAs in various tissues.

			Upstream region		Downstream region	
			Antisense RPKM	Sense RPKM	Antisense RPKM	Sense RPKM
Cerebellum vs Heart (mouse)	Cerebellum-specific pancRNA-bearing genes	cerebellum	96.5	29.2	14.8	410.6
		heart	2.5	11.1	2.1	192.2
	Heart-specific pancRNA-bearing genes	cerebellum	5.2	9.1	3.7	203.3
		heart	144.8	16.7	7.7	486.1
Cerebral cortex vs Cerebellum (mouse)	Cerebral cortex-specific pancRNA-bearing genes	cerebral cortex	74.6	16.1	7.8	304.3
		cerebellum	4.9	15.1	5.3	201.5
	Cerebellum-specific pancRNA-bearing genes	cerebral cortex	4.5	16.0	1.3	220.8
		cerebellum	70.5	21.8	7.8	302.6

The values in this table were normalized by the number of genes. In this analysis, we defined a pancRNA whose RPKM was higher than 0.3 in one tissue and lower than 0.1 in the other as a tissue-specific pancRNA.

Supplementary Table S6

Pearson correlation coefficient between the expression level of pancRNA and the corresponding mRNA

Loci	Pearson correlation coefficient	P-Value
Sh3rf3	0.85	4.95×10^{-6}
Vwa5b2	0.93	7.81×10^{-9}
Pacsin1	0.81	3.13×10^{-5}

Supplementary Table S7

The bias of the pancRNA-bearing protein-coding genes for CpG islands in various chimpanzee tissues

	Candidate pancRNA-bearing genes
	With CpG islands in their promoter regions[a]
Cerebral cortex	94.7%
Cerebellum	93.4%

[a]The percentage of pancRNA-bearing protein-coding genes harboring CpG islands in their promoter regions

Supplementary Table S8

The percentage of genes expressed in various chimpanzee tissues with both "CCGCCG" and "CGGCGG" sequences

All genes	Candidate pancRNA-bearing genes	
	in cerebral cortex	in cerebellum
36.8%	61.2%	60.9%

Supplementary Table S9

Primers for strand-specific RT-PCR analysis

Locus	Name	Primer sequence
Pacsin1	a	CGATCTGATAAGCCCCTTCGTAA
	b	AGCTGCTGCCTTGTATTCTAACT
	c	ATCCCTCGGTTCCCTGACTAATA
	d	CTTGAGAGACCTGGATGTTCACA
	e	CTTCTCTCACCTCCGGATCTCTC
	f	GGAGACTCTTTGGTGGACTCTAT
	g	GGAGAAGTATGAGAAGGTGCTGG
	h	AGATGTGACATTGGGTTAAGTCC
	q	CGCTCCTAATACCTGCTTCTCAT
	r	ATGAGAAGCAGGTATTAGGAGCG
	s	CTTCCTCCTTCCCGCAGC
Kcnmb4	i	GTTTTTCCAAGTCCTGCAGCC
	j	TGCGAAGCGGGAATCTTCAA
	k	GCCAGCTAAGGGTGGCAATA
	l	CGTGCAGGCTAACACGATTG
	m	ACGTGAACAACTCCGAGTCC
	n	CCAACTGTGCCTGTTTCTGC
	o	GATCGGTTCCCAGCCATTCA
	p	ACCACGATGAGAACACCCAC
	v	GAGCTGCTCTCGGAATCCTC
	w	AGGAGCAGCCTCGCTCAA
	x	TTCGAGTGCACCTTCACCTG
	y	GGAGTTGGACTCGGAGTTGTT

Supplementary Table S10

shRNA sequences

Target	Sequence
pancSh3rf3	T**GCAGATTCTCCTAAGCCATGT**TTCAAGAGA**ACATGGCTTAGGAGAATCTGC**TTTTTTGGAAC
pancVwa5b2	T**GATATGAACAAATACTAAAGA**TTCAAGAGA**TCTTTAGTATTTGTTCATATC**TTTTTTGGAAC
pancPacsin1	T**GCTGCTGCCTTGTATTCTAAC**TTCAAGAGA**GTTAGAATACAAGGCAGCAGC**TTTTTTGGAAC

Target sequence in BOLD

Supplementary Table S11

Primers for quantitative RT-PCR analysis

Target		Primer sequence
Sh3rf3	Forward	CATCTGTCCTGCACTGTCCC
	Reverse	ACTTTGGGAGACATGCCCTG
pancSh3rf3	Forward	CAGCCCTTAGCCTGTAGTCC
	Reverse	GAGAGGGTCCAGAATGCCTG
Vwa5b2	Forward	CCAGAGGAGGTGTTATCCGC
	Reverse	GTCAGAGCTTCCATACTCTGCT
pancVwa5b2	Forward	GGGAAGTGAGCGAAGGTAAGT
	Reverse	AGTTCTGACTGCTCCCACCT
Pacsin1	Forward	TGATGGTGTCCGGTGCTC
	Reverse	TTGTAGTTCCCCACCTCCCA
pancPacsin1	Forward	TGCATGCAGACGCTTGTATTG
	Reverse	TTAGTCAGGGAACCGAGGGA
Gapdh	Forward	TCCACCACCCTGTTGCTGTA
	Reverse	ACCACAGTCCATGCCATCAC
