# Supplementary material for: Bidirectional promoters are the major source of gene activation-associated non-coding RNAs in mammals
Source: BMC Genomics. 2014 Jan 17;15:35. doi: 10.1186/1471-2164-15-35 (PMC3898825; doi:10.1186/1471-2164-15-35)

Supplementary Figure S1

The depth of coverage of each base in the mouse genome with reads from directional RNA-seq data of the mouse cerebral cortex. Gene structures are shown in the top of each panel. The depth of coverage with valid reads before and after the removal of sequence duplication is shown at the single nucleotide level in the middle and bottom of each panel, respectively. (A) chr16:27,439,951-27,453,748. (B) chr7:133,970,753-133,973,864. (C) chr3:87,673,360-87,676,342.

Supplementary Figure S2

Either top or bottom strand of genome is preferentially utilized for transcription. Definition of top strand-, bottom strand-, sense and antisense mapped reads. The schematic representation of genomic DNA with a submetacentric centromere (gray circle) is shown in the upper panel. The lower panel shows a magnified representation, in which the black boxes denote exons of protein-coding genes. Filled and open arrowheads represent sense mapped reads and antisense mapped reads, respectively, depending on the strand harboring the corresponding protein-coding genes.

Supplementary Figure S3

(A)(B)(C)(D)(E) Density plots of the ratio of top strand-mapped reads to bottom strand-mapped reads. (F)(G)(H)(I)(J) Density plot of the ratio of top strand-mapped reads to bottom strand-mapped reads on the top and bottom strands of the protein-coding regions, and the intergenic regions.

Supplementary Figure S4

The distribution of sense and antisense mapped reads around the TSS of each gene fraction in the mouse cerebellum, mouse heart, chimpanzee cerebral cortex, and chimpanzee cerebellum. The values in this figure are normalized by the number of genes. (A)(E)(I)(M) All reference genes. (B)(F)(J)(N) The genes with the top 100 most highly expressed pancRNAs (left) and with the 100 most weakly expressed pancRNAs (right), as indicated by RPKM. (C)(G)(K)(O) The distributions in Supplementary Figure S4B, F, J and I were divided into three groups: the genes whose antisense transcript expression level from the upstream region of the TSS was five times higher than that from the downstream region (Condition 1; Left panel). The genes whose antisense transcript

expression level from the upstream region of the TSS was two times lower than that from the downstream region (Condition 2; Middle panel). The remaining genes (Right panel). The values in Supplementary Figure S2C, G, K and O were normalized by the number of genes with pancRNAs showing the top100 ranked RPKM. (D)(H)(L)(P) The genes with the top (left) and the bottom (right) 100 ranked sense RPKMs in the downstream region of their TSSs, respectively.

Supplementary Figure S5

The distribution of longest ORFs in downstream region and upstream region. (A) The mouse dataset. (B) The chimpanzee dataset.

Supplementary Figure S6

The Average RPKM of genes bearing pancRNAs with the 100 top-ranked RPKM and those with the bottom-ranked RPKM relative to RPKM of total genes in all samples. (A) RPKM of the region between +1 and +1,000 bp relative to the TSS as promoter activity for mRNA transcription. (B) RPKM of the mRNA as expression level of mRNA. ***$p < 0.001$; Student's t test.

Supplementary Figure S7

The distribution of sense and antisense mapped reads derived from the mouse cerebellum and heart around TSSs of (A) the cerebellum- and (B) heart-specific pancRNA-bearing genes, respectively. In this analysis, we defined a pancRNA whose RPKM was higher than 0.3 in one tissue and lower than 0.1 in the other as a tissue-specific pancRNA. The values in this figure were normalized by the number of genes.

Supplementary Figure S8

(A)(C)(E)(G) The sequence logos found in the regions from -100 bp to +100 bp and the downstream regions starting from +100 bp relative to the TSS of candidate pancRNA-bearing genes in the mouse cerebellum, mouse heart, chimpanzee cerebral cortex and chimpanzee cerebellum, respectively. (B)(D)(F)(H) The observed frequencies of the "CCGCCG" and "CGGCGG" sequences across the regions around the TSSs of all promoter regions (left) and of candidate

pancRNA-bearing genes' promoter regions (right) in the mouse cerebellum, mouse heart, chimpanzee cerebral cortex and chimpanzee cerebellum, respectively.

Supplementary Figure S1

A

NCBI37/mm9 chr16:27,439,951-27,453,748

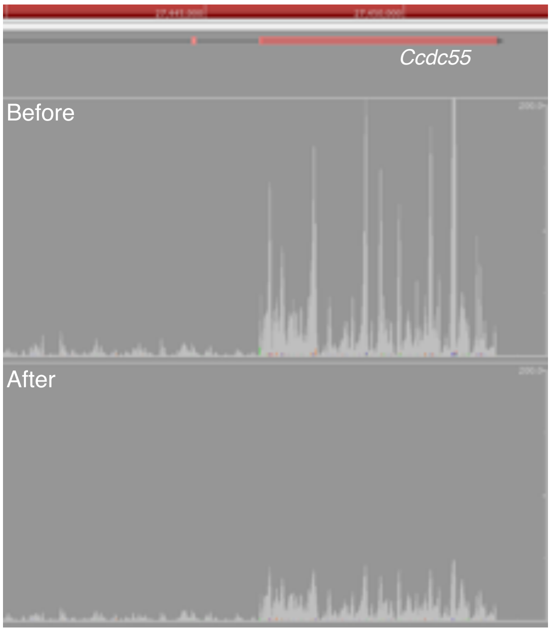

B

NCBI37/mm9 chr7:133,970,753-133,973,864

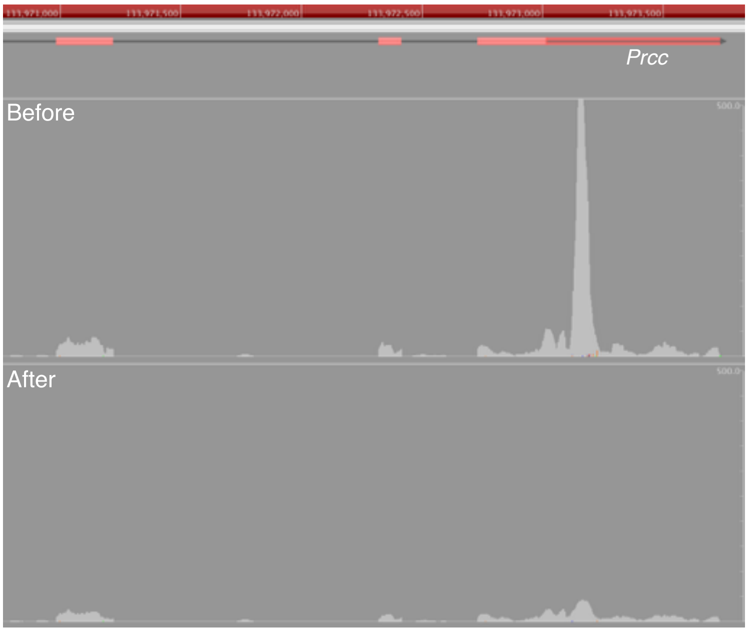

C

NCBI37/mm9 chr3:87,673,360-87,676,342

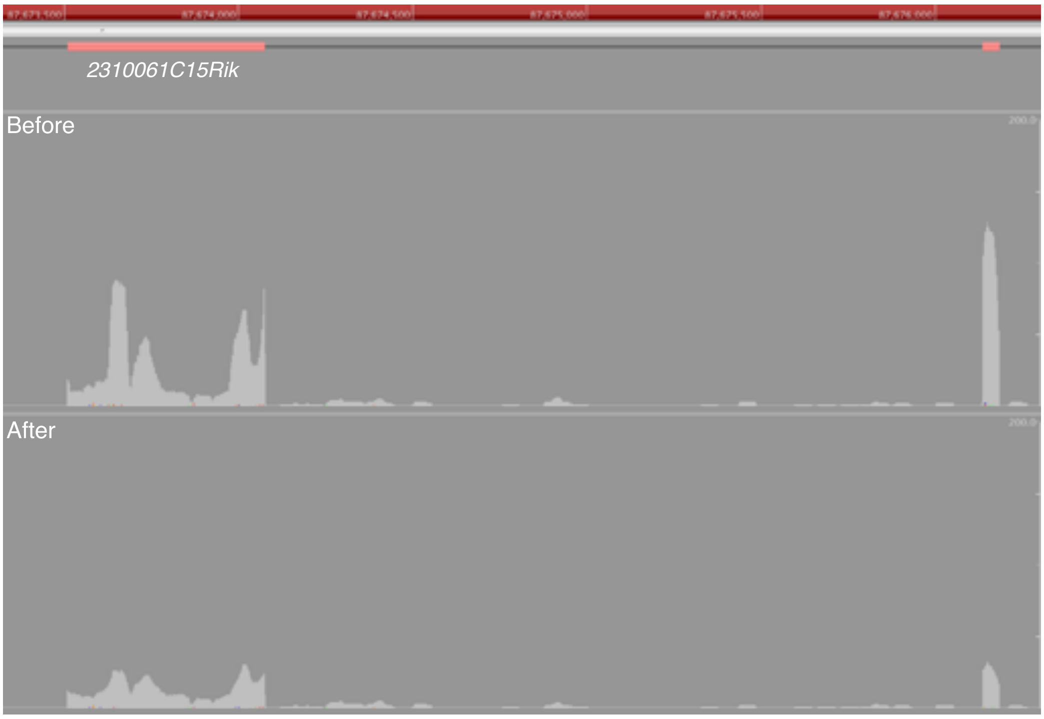

Supplementary Figure S2

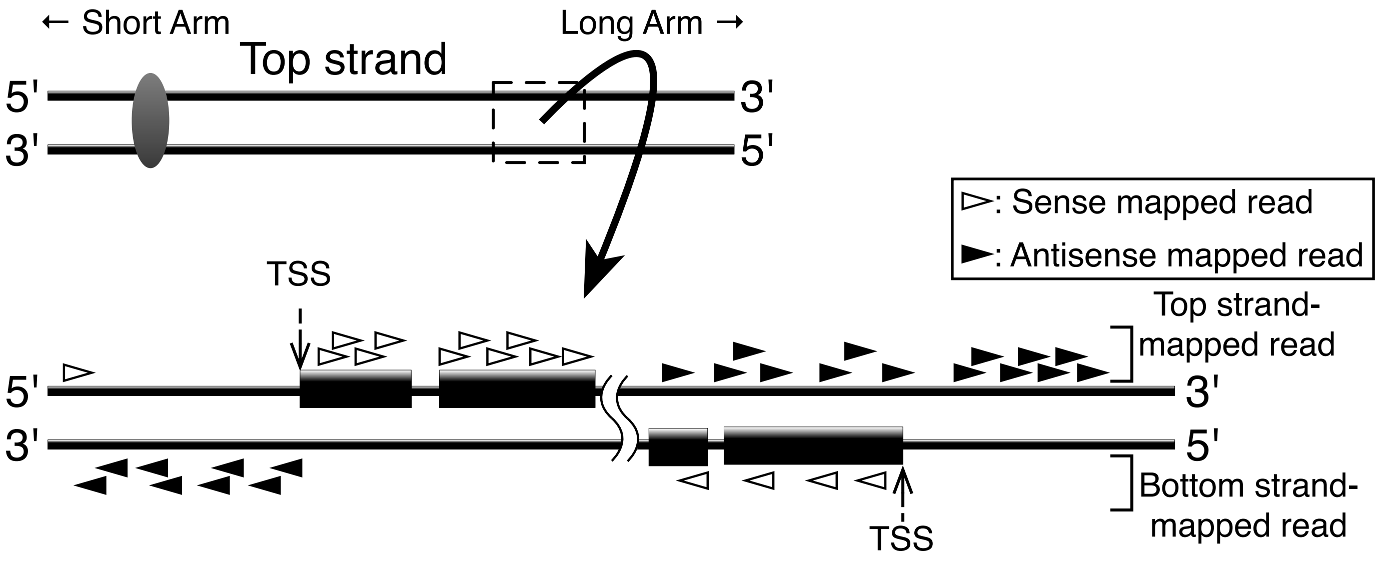

Supplementary Figure S3

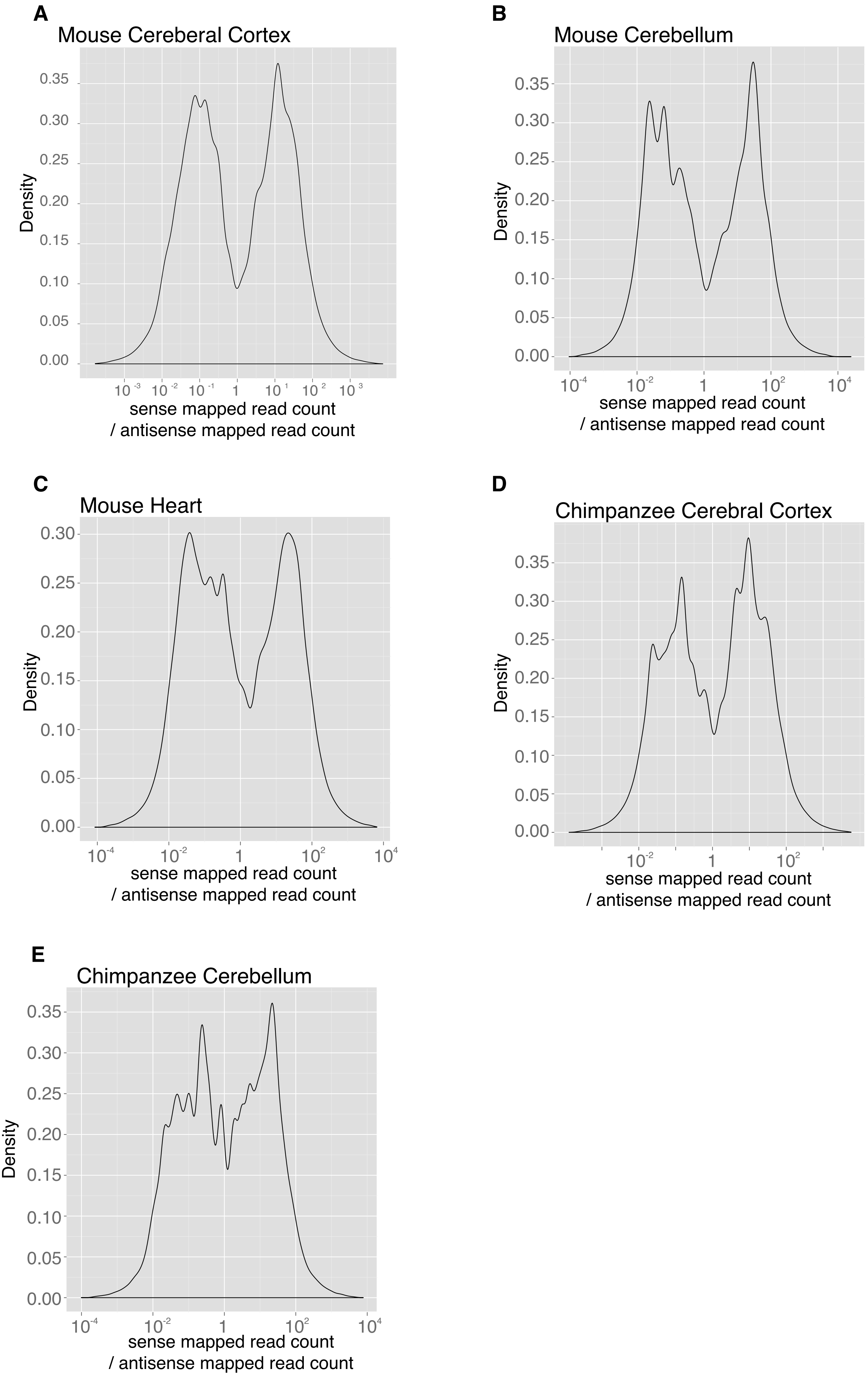

Supplementary Figure S3

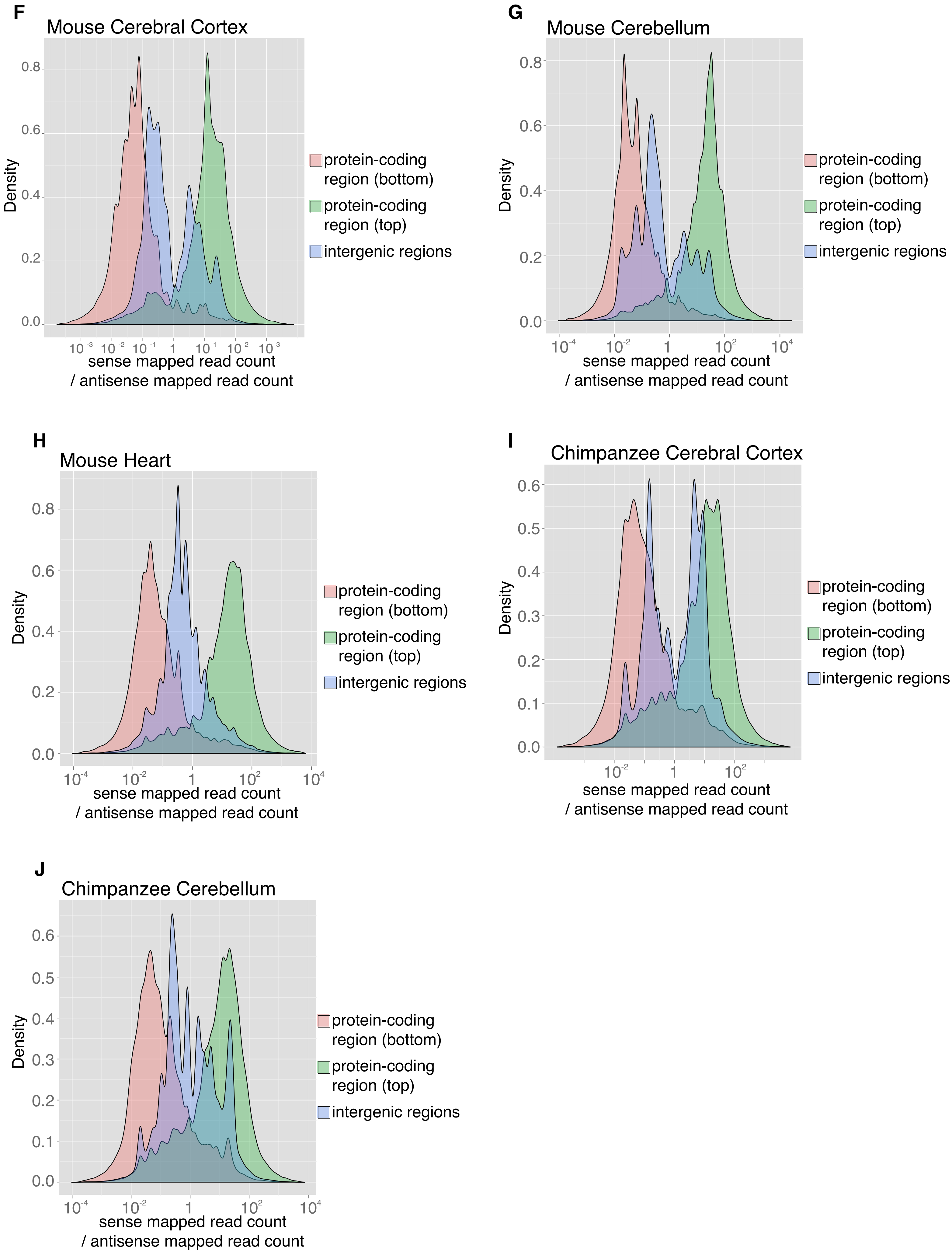

Supplementary Figure S4

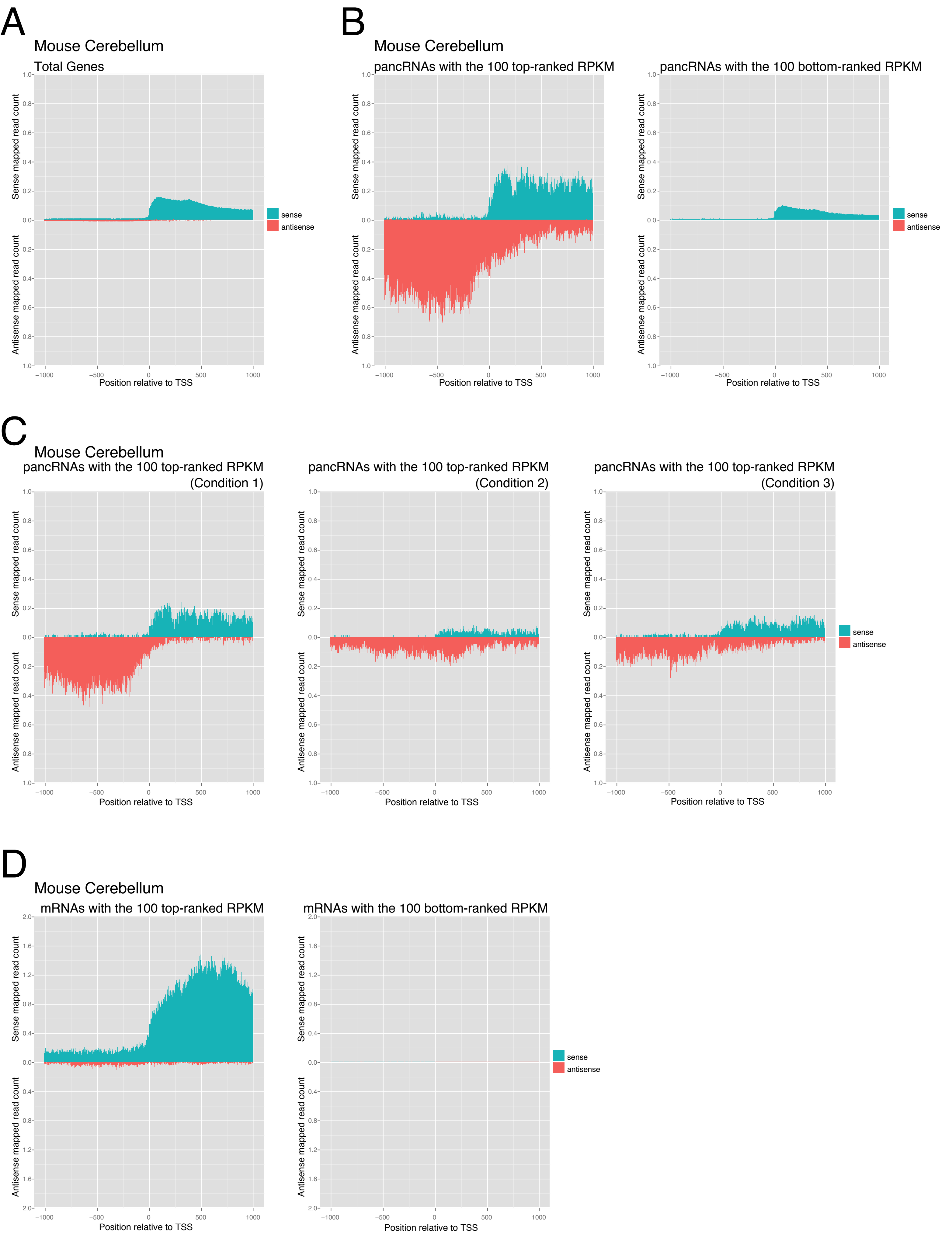

Supplementary Figure S4

E

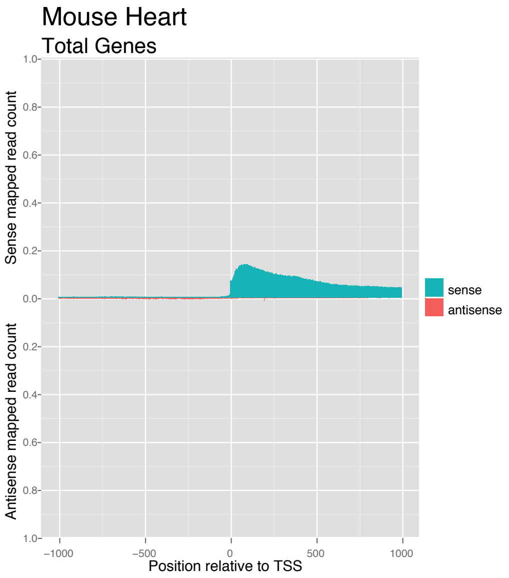

F

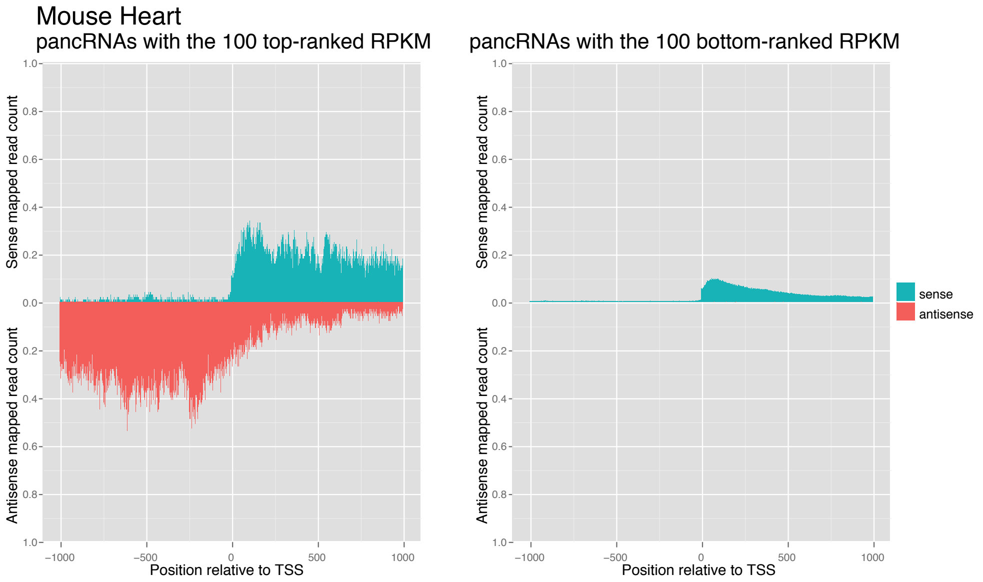

G

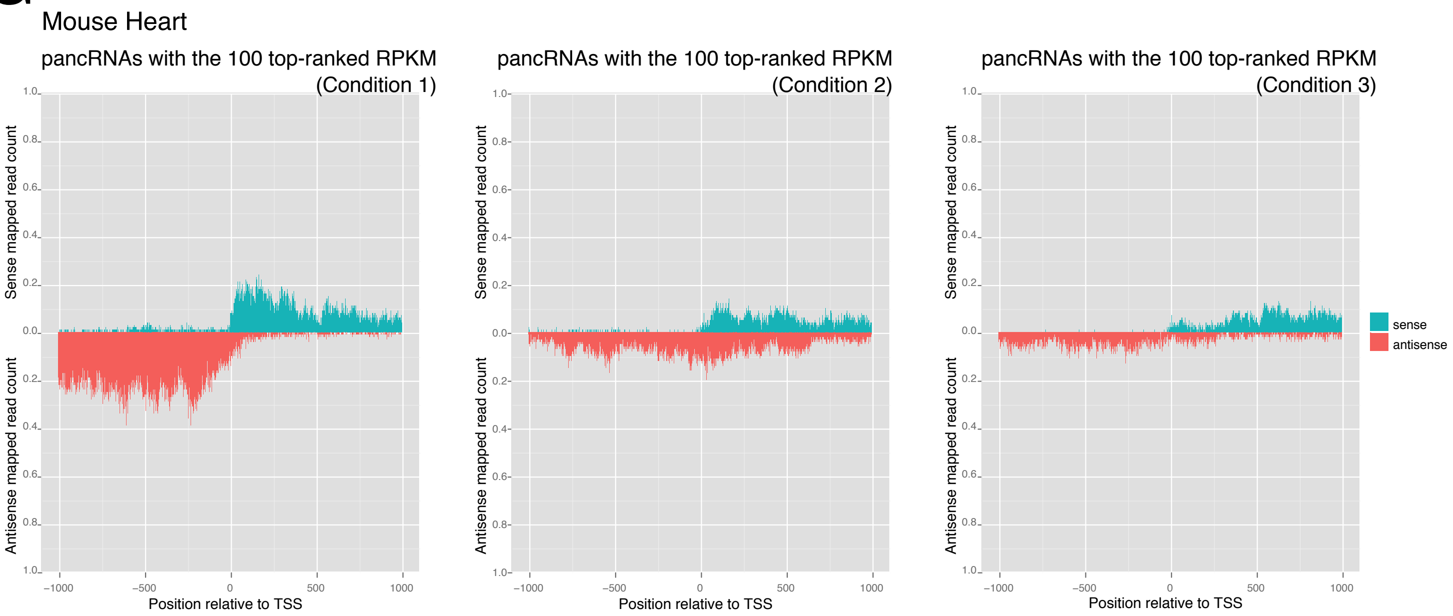

H

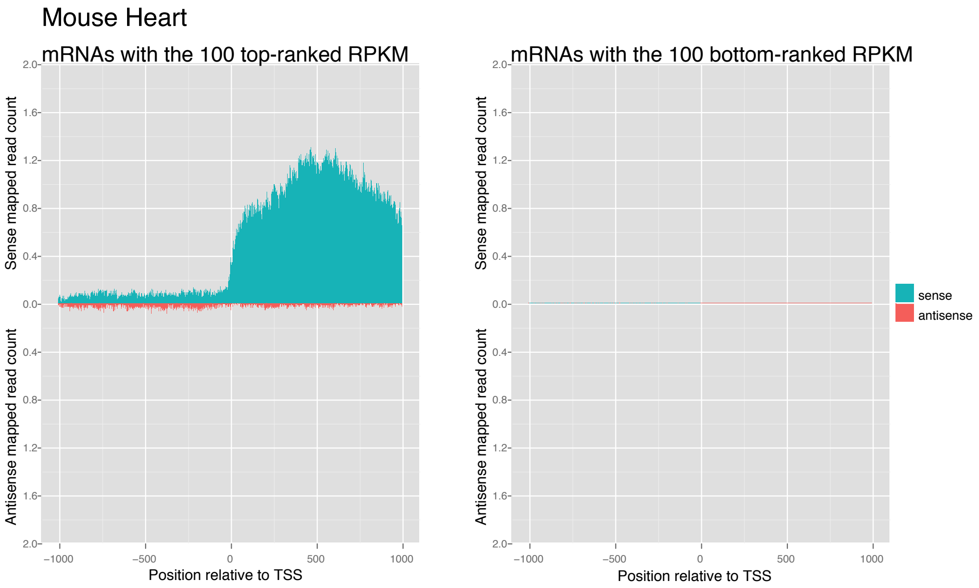

Supplementary Figure S4

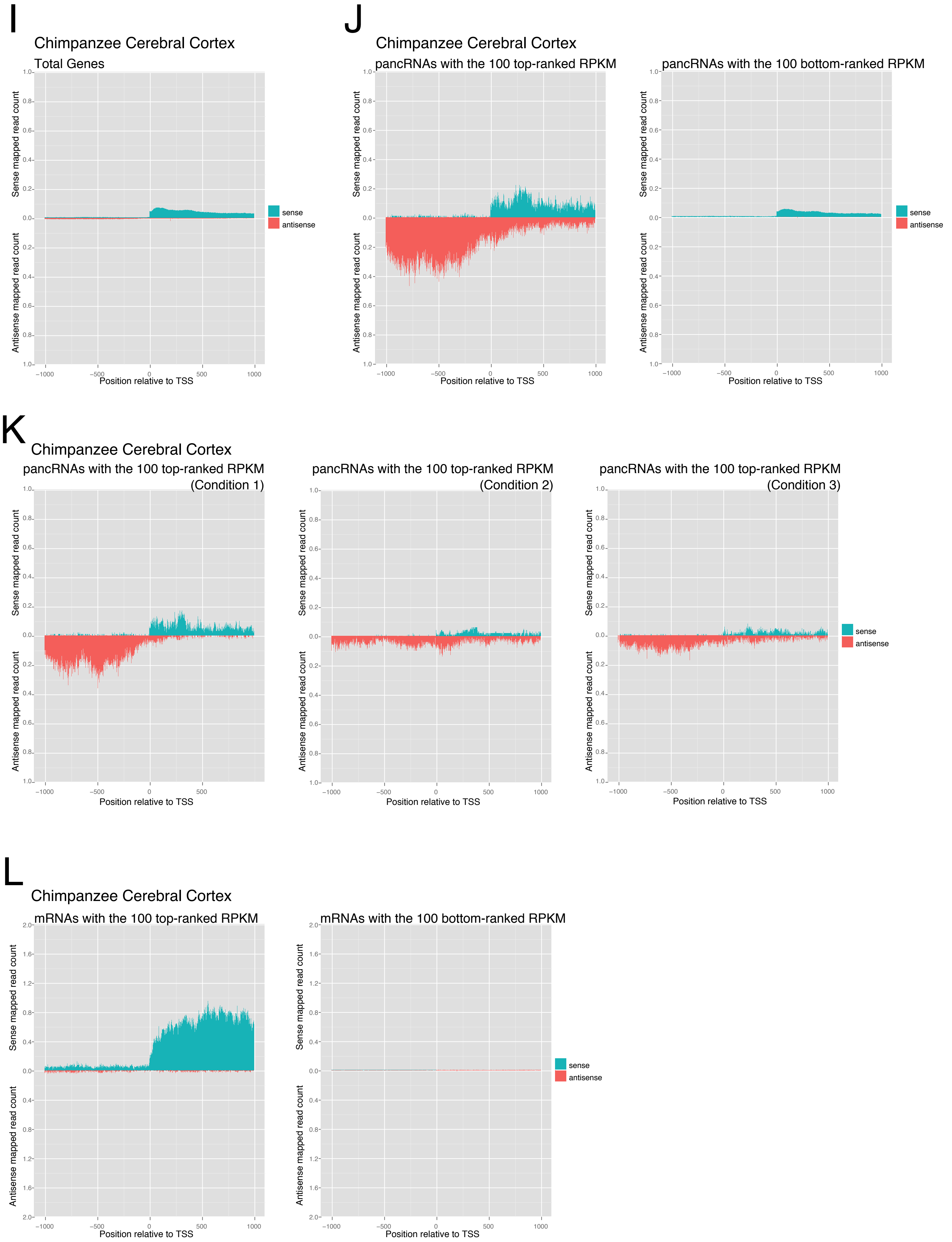

Supplementary Figure S4

M

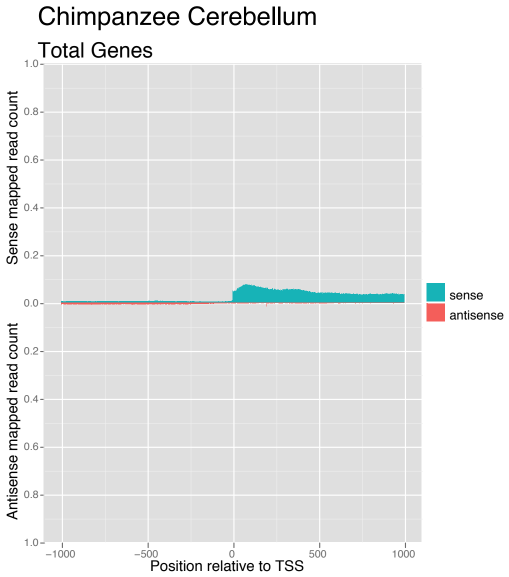

N

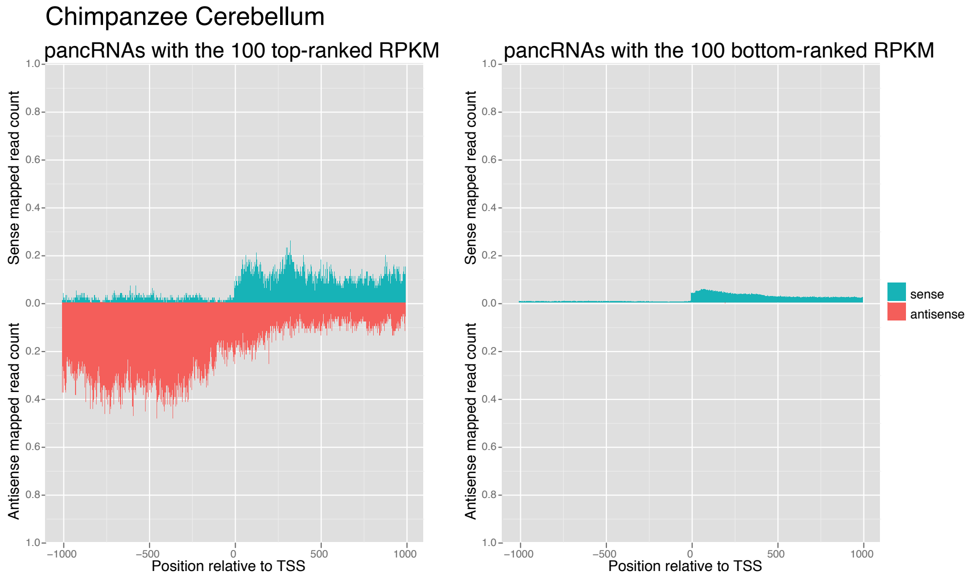

O

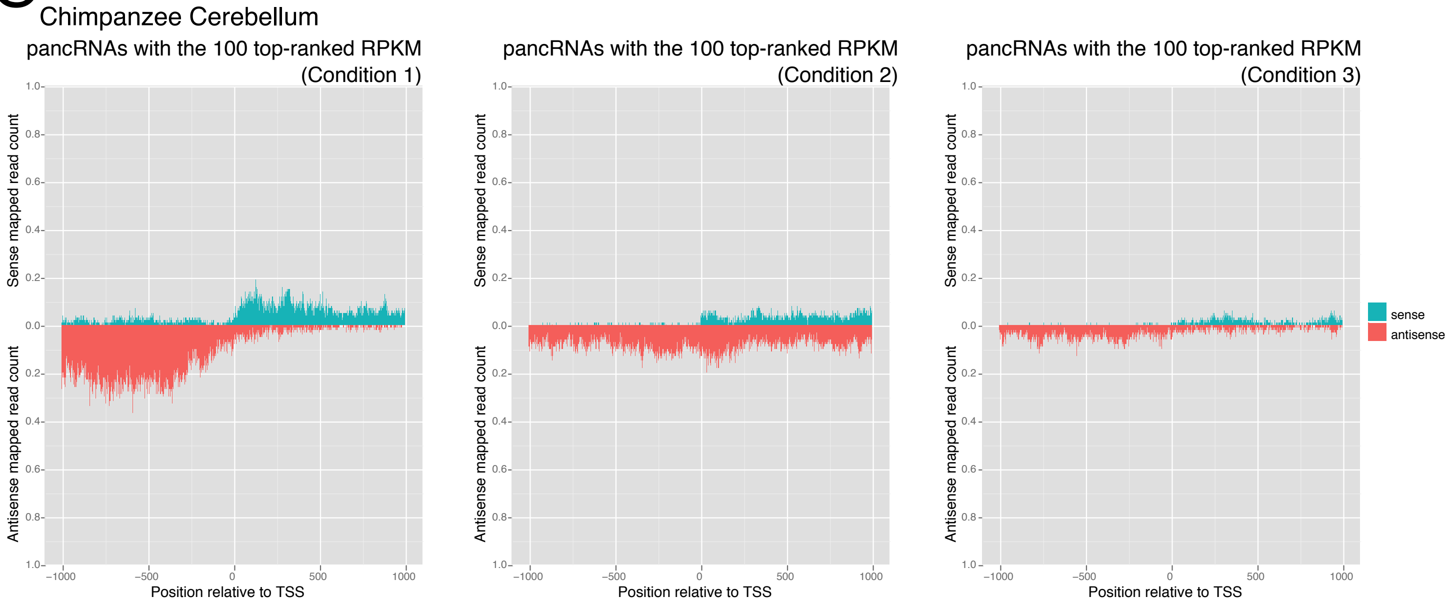

P

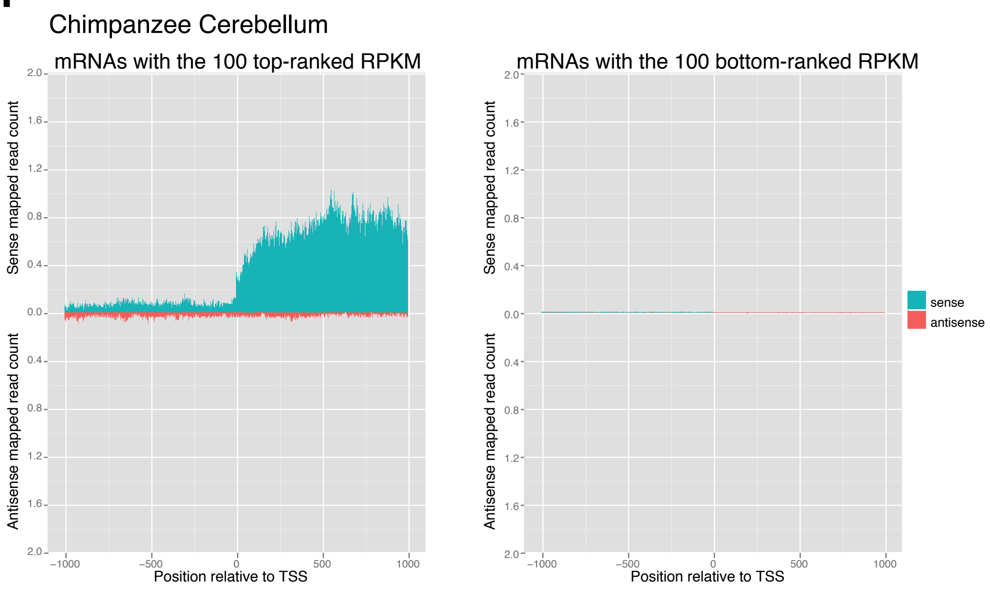

Supplementary Figure S5

A

Mouse Cerebral Cortex

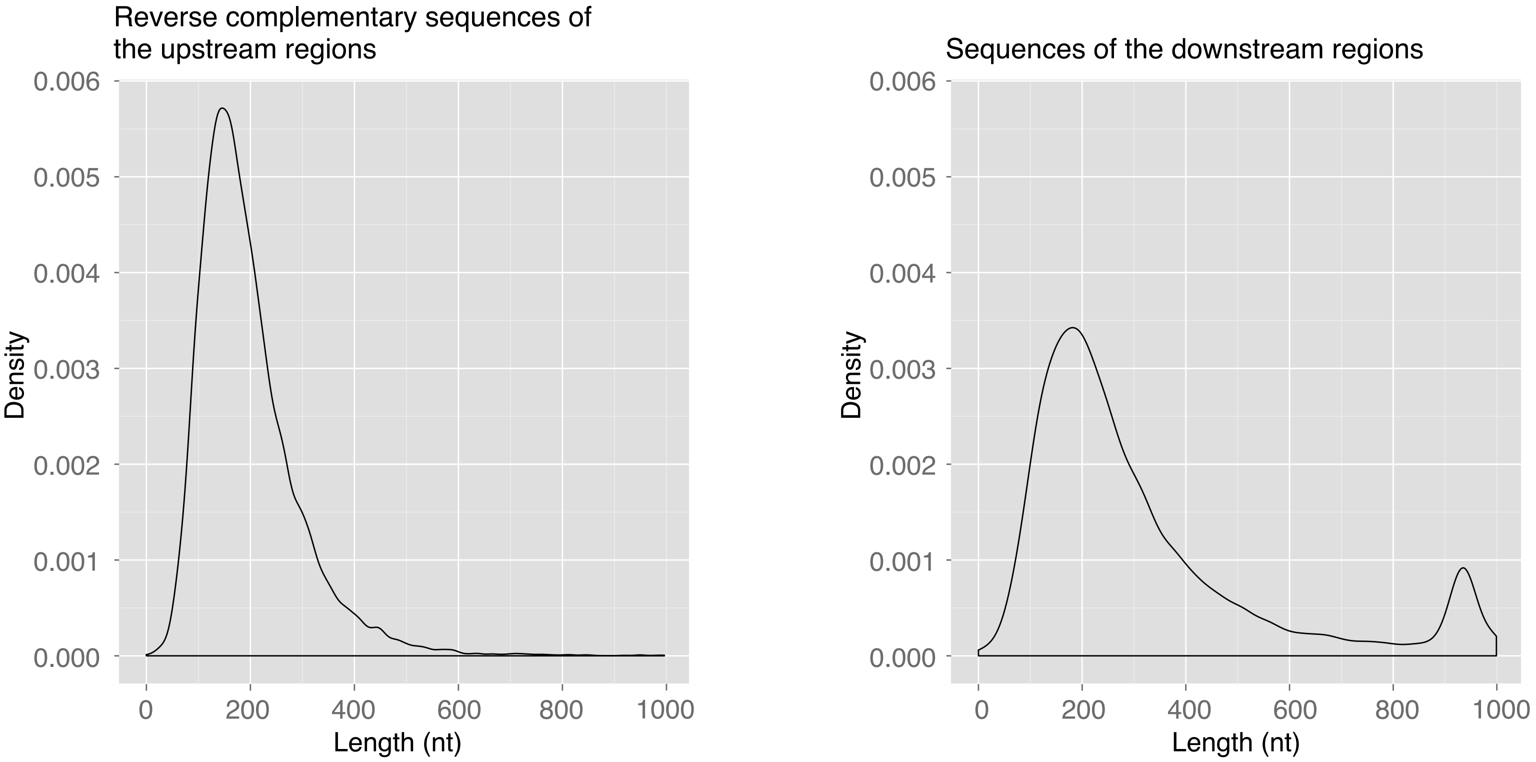

B

Chimpanzee Cerebral Cortex

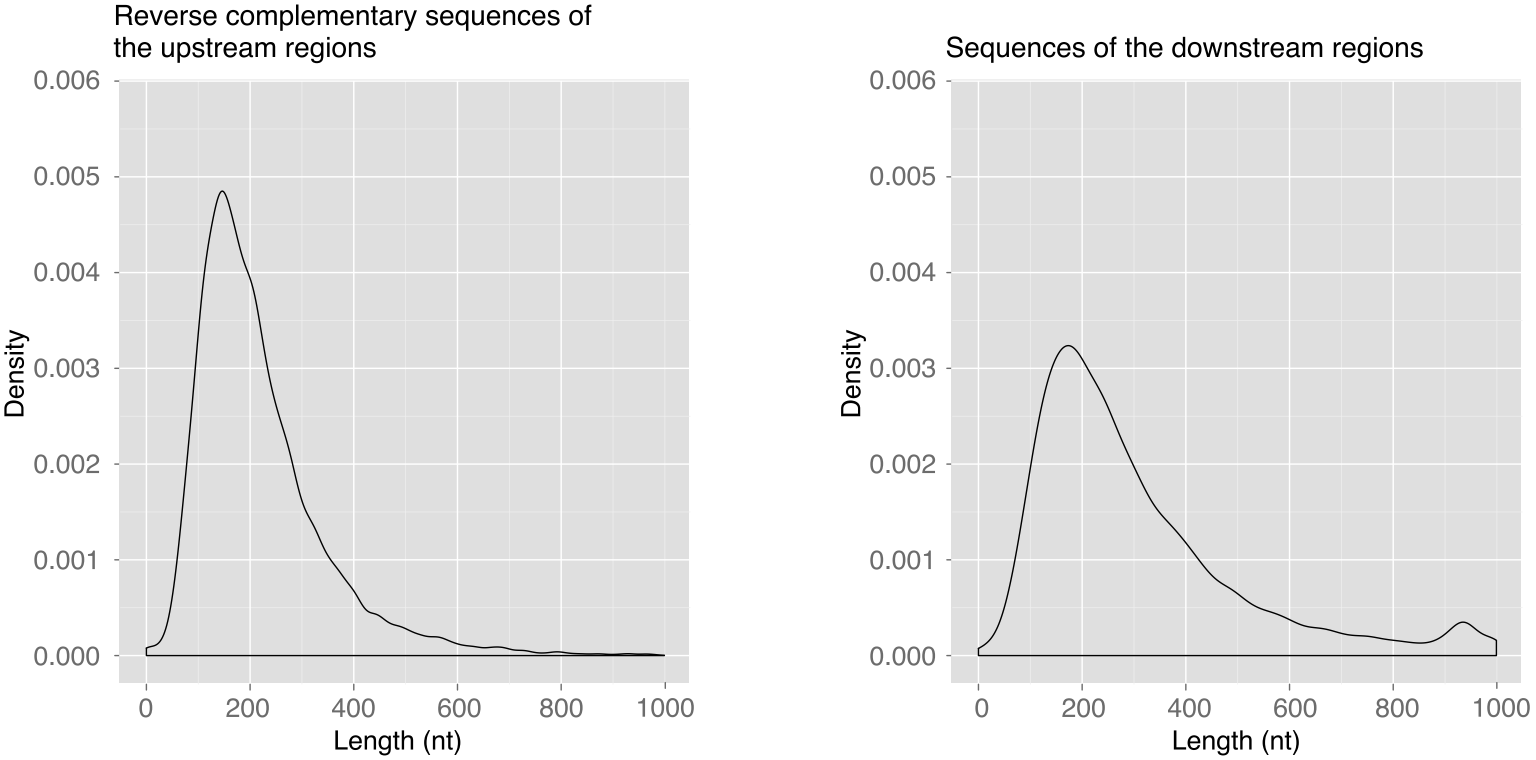

Supplementary Figure S6

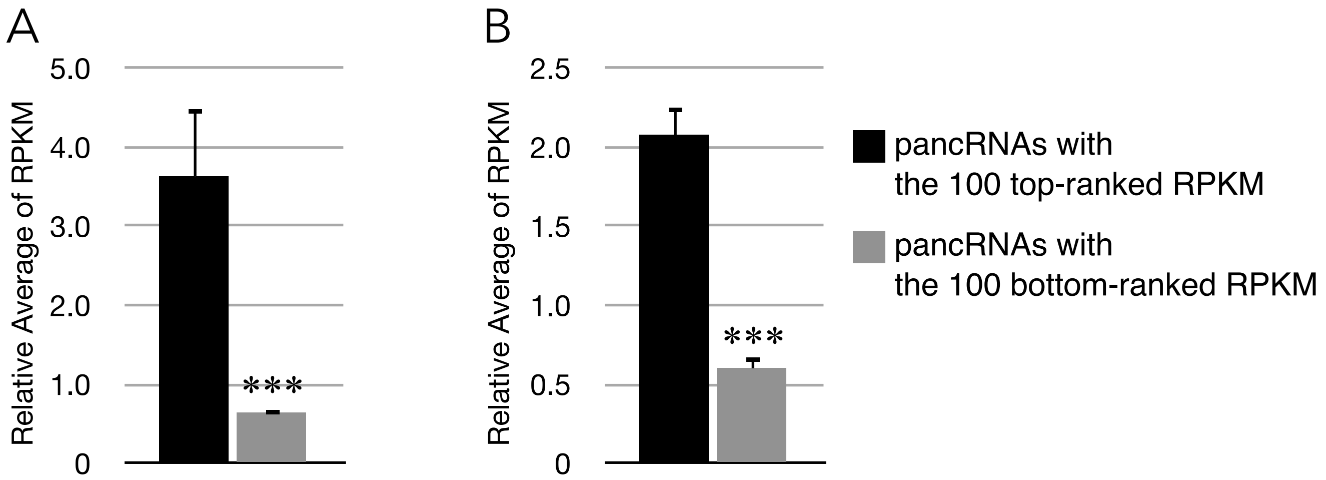

Supplementary Figure S7

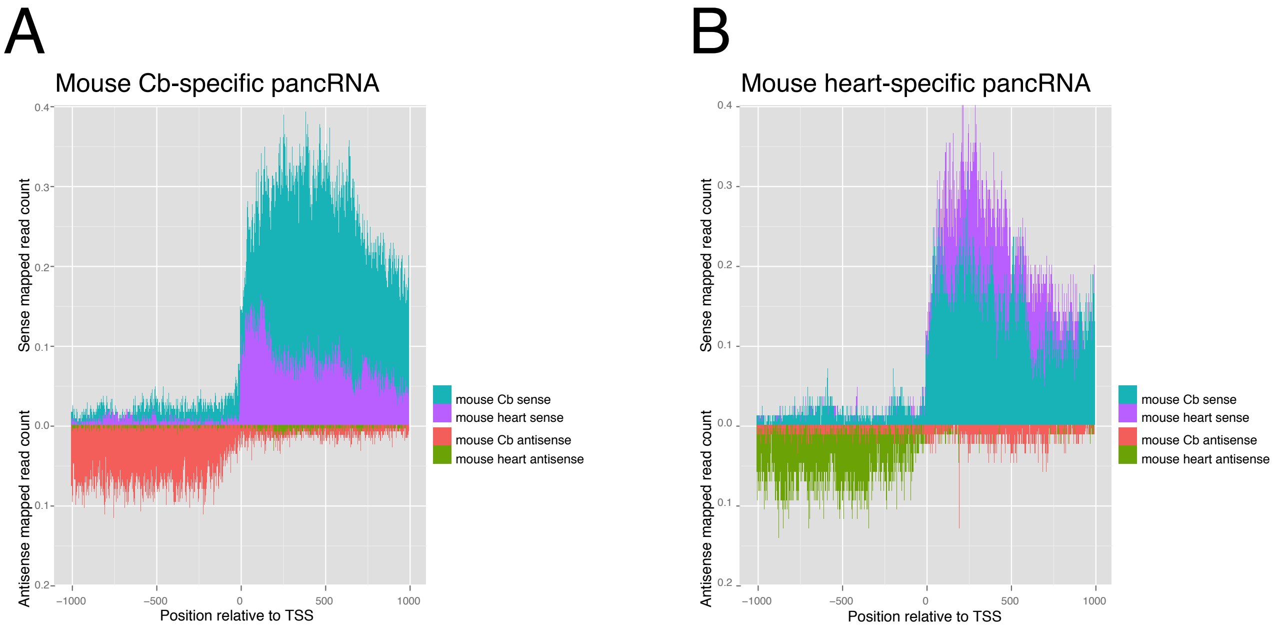

Supplementary Figure S8

A

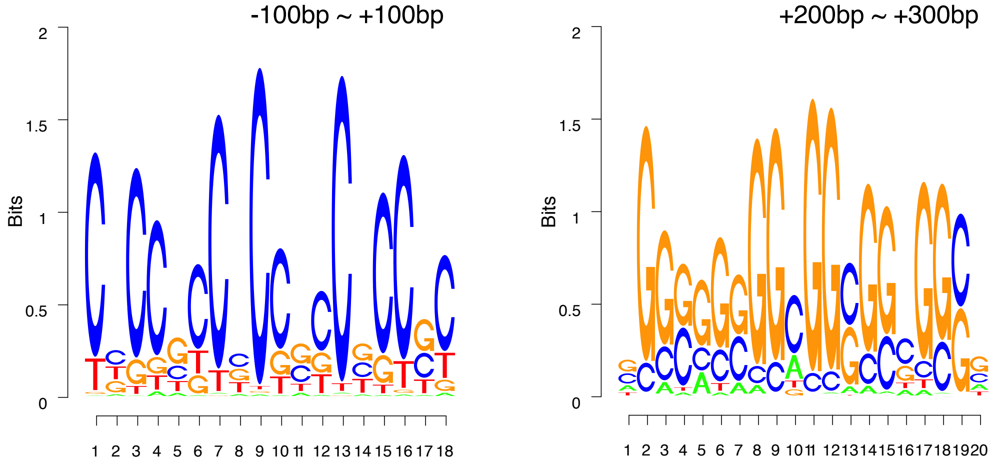

B

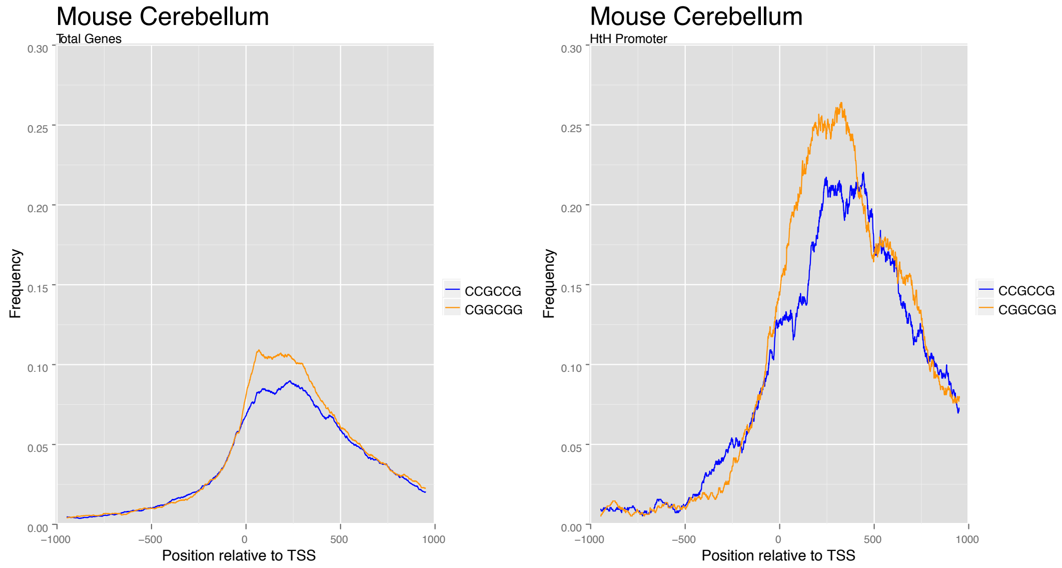

Supplementary Figure S8

C

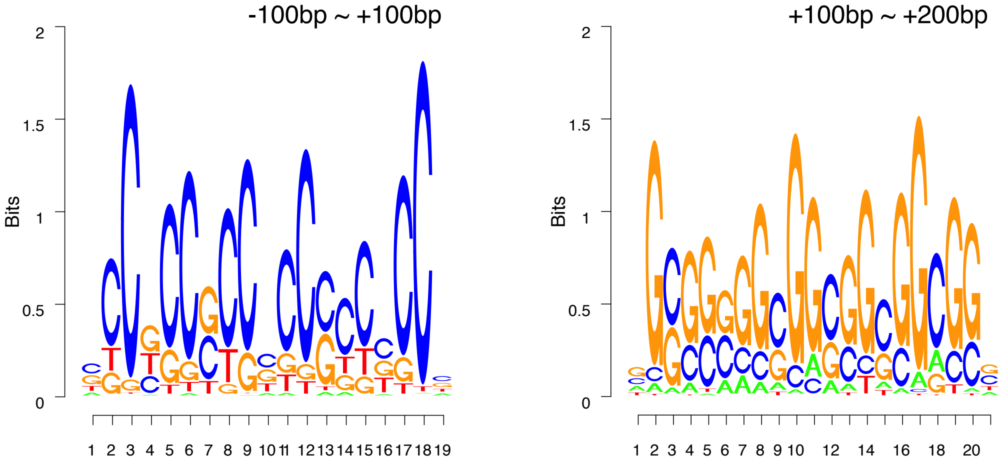

D

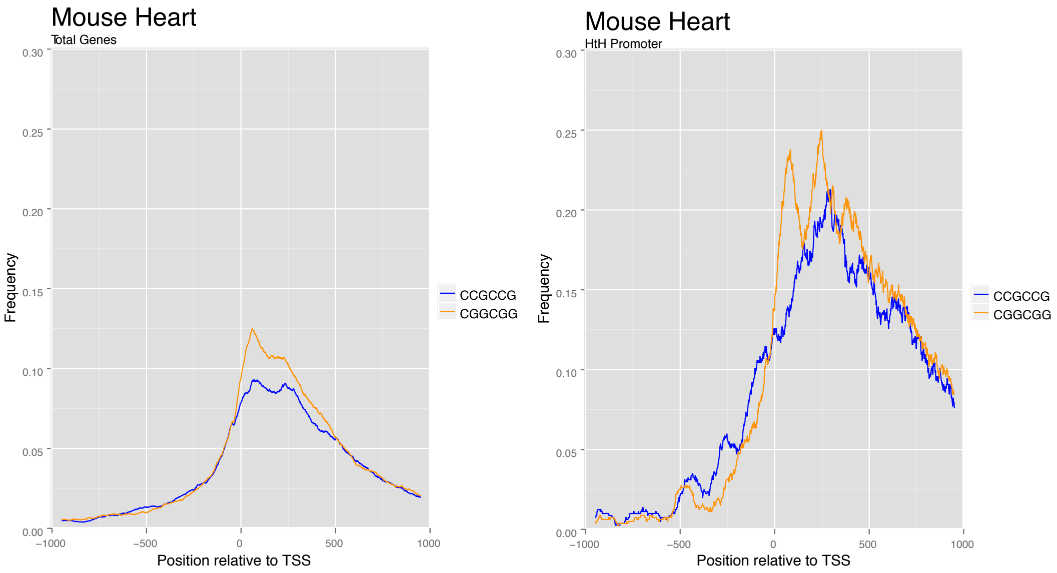

Supplementary Figure S8

E

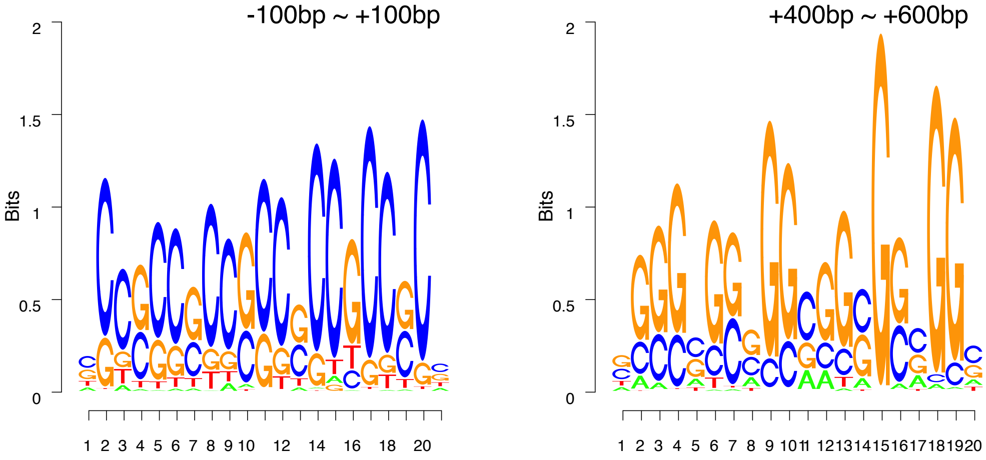

F

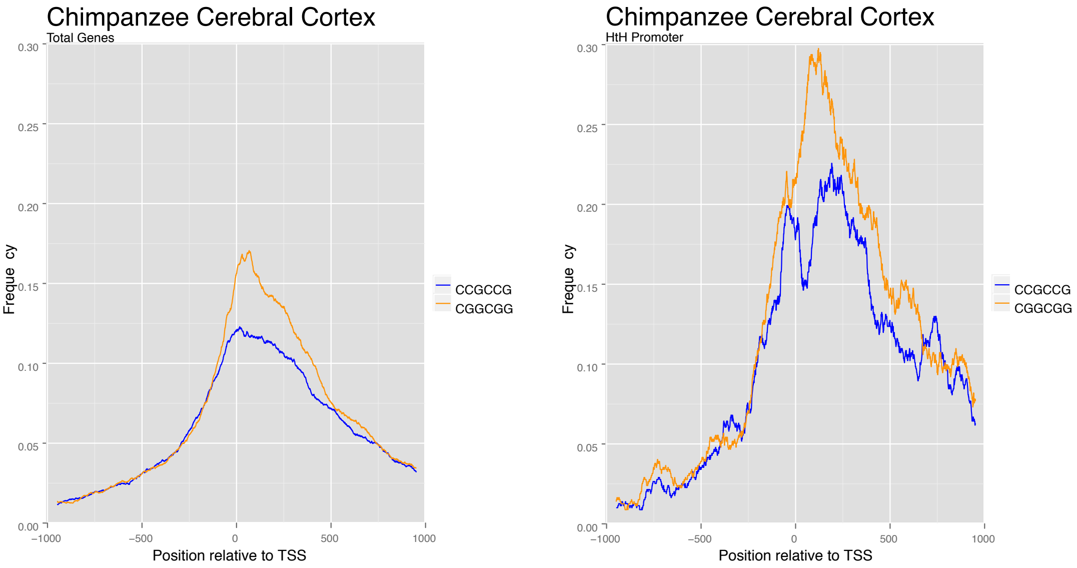

Supplementary Figure S8

G

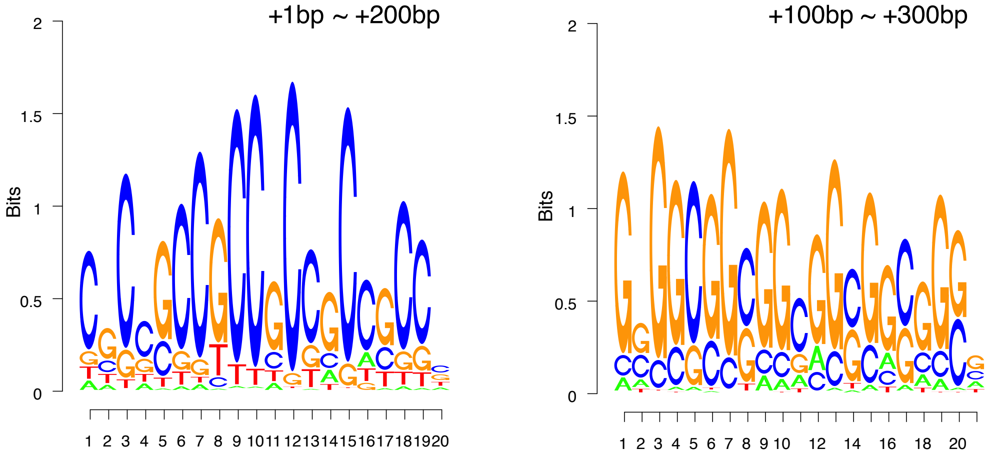

H

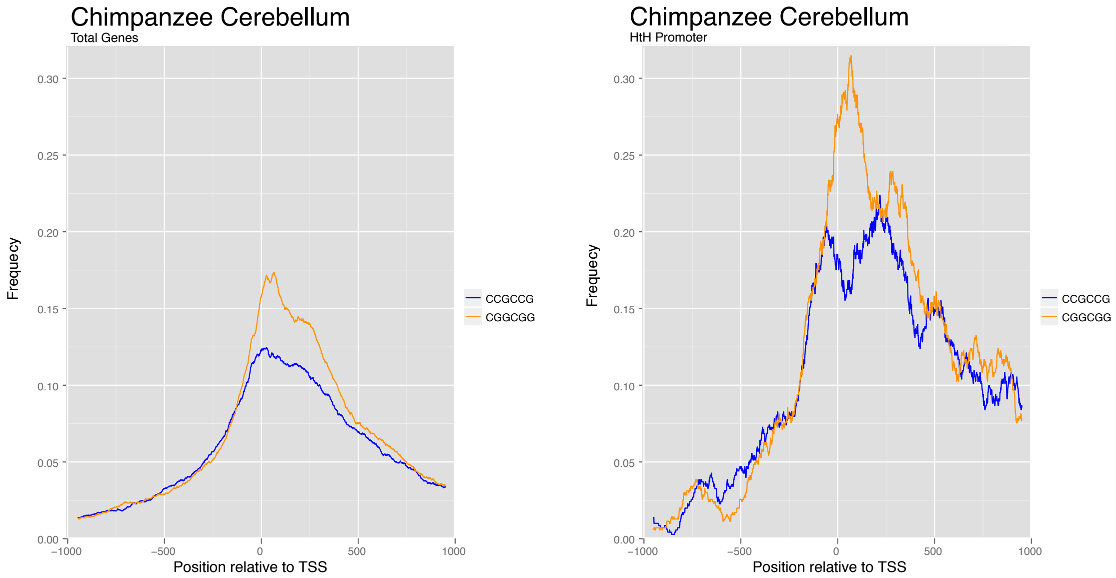

Supplement: Additional file 2 — Figure S1. The depth of coverage of each base in the mouse genome with reads from directional RNAseq data of the mouse cerebral cortex. Figure S2. Either top or bottom strand of genome is preferentially utilized for transcription. Figure S3. Density plots of the ratio of top strand-mapped reads to bottom strand-mapped reads. Figure S4. The distribution of sense and antisense mapped reads around the TSS of each gene fraction in the mouse cerebellum, mouse heart, chimpanzee cerebral cortex, and chimpanzee cerebellum. Figure S5. The distribution of longest ORFs in downstream region and upstream region. Figure S6. The Average RPKM of genes bearing pancRNAs with the 100 top-ranked RPKM and those with the bottom-ranked RPKM relative to RPKM of total genes in all samples. Figure S7. Expression of pancRNAs was accompanied by that of corresponding mRNAs in a tissue-specific manner. Figure S8. Sequence characteristics of pancRNA-bearing genes in the mouse cerebellum, mouse heart, chimpanzee cerebral cortex and chimpanzee cerebellum. [file 1471-2164-15-35-S2.pdf]
